# Ion and Solvent Modulation of Ferrocene and Decamethylferrocene Oxidation Potentials in Organic Electrolytes as Predicted by Molecular Dynamics Simulations

**DOI:** 10.1021/acs.jpcb.4c08321

**Published:** 2025-02-11

**Authors:** John H. Hymel, Suehyun Park, Jesse G. McDaniel

**Affiliations:** School of Chemistry and Biochemistry, Georgia Institute of Technology, Atlanta, Georgia 30332-0400, United States

## Abstract

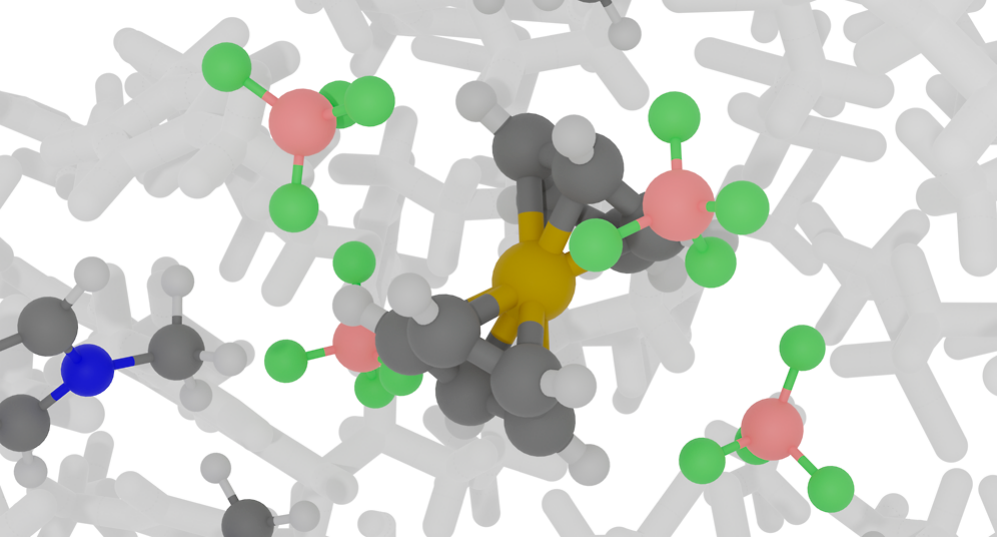

Ferrocene is commonly used as an internal redox couple in electrochemical measurements. Therefore, understanding how the absolute oxidation potential of ferrocene is modulated by different solvents and ion concentrations is important for the comparison of experimental measurements between different electrochemical systems. While standard implicit solvation models may provide relatively good predictions in bulk solvents, they lack the ability to describe ion coordination effects that can substantially alter redox potentials in practical electrolyte systems. In this work, we utilize molecular dynamics simulations to compute absolute oxidation potentials for the ferrocene and decamethylferrocene redox couple in bulk solvents of water, acetonitrile, 1,2-dichloroethane, and trichloromethane, as well as organic electrolytes consisting of mixtures of [BMIM^+^][BF_4_^–^] ionic liquid and acetonitrile and 1,2-dichloroethane solvents, for a wide range of ion concentrations. The goals are twofold: first, for the bulk solvents, we compare and evaluate the consistency of redox potential predictions for polarizable and nonpolarizable force fields from explicit solvent, free energy simulations, with predictions from an implicit solvent model. Second, we evaluate how ion coordination within the organic electrolytes modulates the redox potential of ferrocene and decamethylferrocene as a function of the ionic concentration and solvent dielectric constant. Utilizing linear response theory, we analyze the solvation contribution to the redox potential in terms of distributions of anion coordination number and how the anion coordination modulates the vertical ionization energy. We show that inclusion of liquid-vacuum interfacial potentials is essential for consistent prediction/interpretation of redox potentials across different solvents and force fields in order to compensate for the artificial quadrupole trace contribution to the solute cavity interfacial potential; this important consideration was previously proposed by Harder and Roux [*J. Chem. Phys.***2008**, 129, 234706].

## 1 Introduction

The first-principles prediction of redox potentials^1^ is one of the many goals within computational electrochemistry.^2^ Redox potentials are typically analyzed using a thermodynamic cycle, consisting of the gas-phase ionization potential and difference in solvation free energy of the initial and final charge states of the redox moiety.^1^ Their computation thus requires accurate electronic structure approaches to compute ionization potentials^3−5^ and accurate prediction of solvation free energies;^1,6^ more subtle details such as surface/interface potentials may also need to be considered.^1,7^ In this work, we focus on oxidation potential predictions for the ferrocene and decamethylferrocene (DMFc) redox couples for several solvents and organic electrolytes of systematically varying ion content. The major focus, for comparative purposes, is thus prediction of solvation free energies (and interface potentials) within the different solvents/electrolytes, whereas the gas-phase ionization potentials of ferrocene and DMFc are taken as the National Institute of Standards and Technology (NIST) recommended value.

In electrochemistry, the ferrocene/ferrocenium (Fc/Fc^+^) redox couple has historically served as the recommended internal reference redox system for reporting redox potentials in nonaqueous solvents.^8^ The motivation for employing an internal reference redox couple in combination with a quasi-reference electrode^9,10^ is to avoid liquid junction potentials and/or contamination that would occur when an aqueous reference electrode (e.g., Ag/AgCl/KCl(sat’d)) is used for measurements within nonaqueous electrolytes.^11^ In addition to being a stable/reversible redox standard, the Fc/Fc^+^ redox couple has been proposed as a “universal reference electrode”, potentially enabling comparison of redox potentials measured in different nonaqueous solvents;^8^ the prerequisite being that the redox potential should be nearly solvent independent (“Strehlow assumption”^12^). However, other experiments demonstrate that the oxidation potential of ferrocene is significantly dependent (e.g., variations up to 0.5 V) on both solvent and electrolyte ion concentration, due presumably to differences in solvation energy of the ferrocenium cation.^10,13^

There is much physical basis for why the redox potential of a given moiety should vary among different solvents/electrolytes. Since the redox species will generally be ionic in either (or both) the initial or final state of a redox process, the redox potential has a substantial contribution from ionic solvation energies (an exception is proton coupled electron transfer (PCET) processes,^14^ which are not considered here). Ionic solvation energies are large, typically ranging from ∼2–5 eV for monovalent ions (with an exceptionally higher value for the proton) and are substantially larger for divalent ions.^6^ Differences of ∼0.4–0.5 eV are commonly observed across solvents of differing dielectric strength,^6^ as rationalized by the Born model. In electrolytes, the ionic environment can also substantially alter solvation energies and hence redox potentials. For electrolytes of dilute to moderate ion concentration, redox potentials may be modulated by ion pairing/clustering and other ion correlation effects, particularly in lower dielectric solvents.^15,16^ Alternatively, in concentrated electrolytes such as ionic liquids, ionic solvation energies may be influenced by “overscreening effects” that arise from the specific nanoscale ionic structure of the liquid and are not predicted from continuum properties such as the dielectric constant.^17,18^

Due to its fundamental importance in electrochemistry, there have been many experimental studies dedicated to the characterization of thermodynamic redox potentials (and additionally electron transfer rates^11,19−24^) of ferrocene and related metallocenes in different electrolyte media.^10,12,13,25−27^ We note that while electron transfer kinetics will not be discussed here, ion solvation effects on the redox potential will similarly be relevant to the outer-sphere, reorganization energies that enter Marcus theory rate expressions.^28^ Noviandri et al. measured the redox potential of ferrocene/ferrocenium (Fc/Fc^+^) in 29 different solvents, utilizing the larger decamethylferrocene/decamethylferrocenium (DMFc/DCFc^+^) couple as a redox standard.^25^ For nonaqueous solvents, the redox potential of Fc/Fc^+^ was found to vary by ∼150 mV vs DMFc/DCFc^+^ across the different solvents; water showed anomalous behavior relative to the derived solvation model fit to the nonaqueous solvent data.^25^ Bao et al. investigated ion concentration effects on the oxidation potential of ferrocene for supporting electrolyte (tetrabutylammonium/tetrafluoroborate) concentrations ranging from 1 to 500 mM in dichloromethane, acetonitrile, and dimethylformamide solvents.^13^ The authors observed concentration-dependent, redox potential shifts up to ∼500 mV, with the most extreme shifts in the low dielectric, dichloromethane solvent.^13^ The fact that ion pairing effects may substantially modulate the oxidation potential of ferrocene was previously proposed by Hupp, based on charge-transfer experiments on an acetylene-bridged biferrocene monocation.^15^ From such experiments within BF_4_^–^, PF_6_^–^, and ClO_4_^–^-based electrolytes in acetonitrile, nitromethane, acetone, or dichloromethane solvent, redox potential shifts were estimated up to 200 mV for 100 mM concentration electrolytes and 60 mV for 1 mM concentration electrolytes, with the largest shifts for low-dielectric, dichloromethane solvent.^15^ Finally, and as relevant to this work, redox potentials of ferrocene have been measured in room temperature ionic liquids.^26,29−31^ While differences of hundreds of mV in the ferrocene redox potential within various ionic liquids are observed compared to molecular organic solvents,^26^ the solvent effect is difficult to interpret due to the unknown liquid junction potential formed at the ionic liquid and reference electrode interface.

These empirical data clearly indicate substantial variation of the redox potential of ferrocene within different electrolytes and thus that the “Strehlow assumption”^12^ is inappropriate in general.^10^ In this regard, computational approaches are very useful tools for predicting absolute redox potentials and interpreting the contribution of various solvent (and ion) effects within different electrolytes. In a recent work, Makos et al. presented a computational study for the first-principles prediction of absolute oxidation potential of ferrocene within bulk acetonitrile.^32^ These authors employed sophisticated electronic structure theory methods, consisting of equation-of-motion ionization potential coupled-cluster singles and doubles (EOM-IP-CCSD) paired with effective fragment potentials (EFP), achieving highly accurate predictions for the absolute oxidation potential of ferrocene in acetonitrile solvent as compared to the recommended experimental reference value. To our knowledge, similar high-quality computational predictions of redox potentials for ferrocene within concentrated organic electrolytes do not exist, and this is the goal of this work (albeit at a different level of theory). We note that there have been computational studies of electron transfer rates that involve related reorganization energy calculations for ferrocene/ferrocenium for both homogeneous case^33^ and heterogeneous electron transfer at electrode/organic electrolyte interfaces;^34,35^ in this regard, benchmarking thermodynamic redox potential predictions in such electrolyte systems is an important endeavor.

For computational redox potential predictions, it is important to consider the choice of different methods and levels of theory. While implicit solvent approaches often provide fairly good predictions for redox potentials in bulk solvents,^1^ in general, they do not capture ion pairing/correlation effects relevant to the organic electrolytes studied here and thus will not be considered. Quantum mechanics/molecular mechanics (QM/MM) is a powerful approach in principle, as it can explicitly capture the electronic ionization process while describing solvent interactions in atomistic detail. QM/MM approaches have been employed to predict redox potentials of metallocenes,^32,36^ transition-metal complexes,^36^ phenol and its derivatives,^4,37,38^ polyoxometalates,^39^ and other small organic molecules,^36−38,40^ with most of these studies focusing on aqueous solvents (or acetonitrile^32^). However, there are some limitations to QM/MM for redox potential prediction. Most QM/MM studies employ linear response approximations,^4,32,36,40^ which have been shown to be quite accurate for bulk solvents but may incur error, for example, describing ion pairing effects in organic electrolytes (as shown in this work). Also, due to computational expense, the required ensemble of solvation structures must often be generated at a lower level of theory [e.g., classical molecular dynamics (MD)] than utilized for the QM/MM energy evaluations;^4,32,40^ note that this issue may be overcome with free energy perturbation or free energy path methods.^41^ Finally, it has been shown that there is very slow *R*^–1^ convergence of the predicted ionic solvation free energy with the electrostatic embedding cutoffs employed in QM/MM calculations.^40^ Ab initio molecular dynamics (AIMD) is an alternative approach that has been utilized for redox potential predictions of numerous systems,^42−47^ in which both the redox moiety and solvent are modeled at a quantum mechanical level of theory [typically density functional theory (DFT)]. AIMD may be particularly advantageous for studying transition metal complexes that exhibit changes in “inner-sphere” ligand/solvent coordination during a redox process, for which a force field description (e.g., as in QM/MM) would be inappropriate. If the solution-phase, redox process is reversible and does not involve chemical transformation, then a thermodynamic cycle can be utilized in which solvation is separated from the ionization process, so that purely force field-based, classical MD can be utilized to predict the solvation energetics.^1^ This is the approach employed in this work, since classical MD enables the long simulation times (e.g., hundreds of nanoseconds) required for statistical sampling challenges associated with both low concentration (e.g., ion pairing/correlation effects) and high concentration (e.g., highly viscous) organic electrolytes.

The accuracy of solvation free energy predictions for redox potentials with classical MD will depend on the employed force field. In prior computational work,^36−39^ organic redox moieties and common solvents have been modeled with standard force fields such as OPLS-AA, GAFF, and/or specialized water force fields such as SPC/E or TIP3P;^48^ however, the availability/accuracy of a force field becomes more of a concern when modeling solutes such as transition metal complexes with strong/directional solvent interactions^36^ and/or organic electrolytes or ionic liquids for which explicit incorporation of electronic polarization has been deemed essential.^49,50^ In some cases, ab initio-based force fields and/or EFPs have been utilized for the solvent description in redox potential predictions,^4,32,40^ and in other cases, polarization has been incorporated with Drude oscillator models.^36,38^ While the prediction of solvation free energies for neutral organic substrates in aqueous systems has been well-benchmarked for conventional force fields,^48^ the prediction of ion solvation energies across a variety of solvent/electrolyte systems (i.e., as required for redox potentials) has not. For such a benchmark/comparison, as is done in this work, it is essential to consider the subtle aspect of force field prediction of interfacial potentials, for which differences of hundreds of mV can result for a given system.^7,51^ As postulated by Harder and Roux,^7^ it may be essential to incorporate solvent/vacuum interfacial potential calculations to cancel force field artifacts associated with the solute cavity interfacial potential, which to our knowledge is not commonly done in recent literature on first-principles, redox potential prediction.

In this work, we present absolute redox potential predictions for ferrocene and DMFc in a variety of organic solvents and organic electrolytes of varying ion concentration. Specifically, we investigate water (H_2_O), acetonitrile (ACN), 1,2-dichloroethane (DCE), and trichloromethane (TCM) bulk solvents and organic electrolytes composed of 1-butyl-3-methylimidazolium tetrafluoroborate ([BMIM^+^][BF_4_^–^]) ionic liquid and ACN or DCE solvent mixtures for systematically varying ion concentration up to the pure ionic liquid limit (100% ions). For the bulk solvents, redox potentials (i.e., solvation free energies) are predicted from classical MD simulations utilizing nonpolarizable OPLS-AA force field^48^ and polarizable SAPT-FF force field,^16,52^ the latter being an ab initio force field previously developed on the basis of symmetry-adapted perturbation theory (SAPT)^53^ and extended to ferrocene in this work. Naive prediction with classical MD without consideration of interface potentials suggests dramatic differences between the two different force fields compared to implicit solvent models. However, properly accounting for the artificial force field contribution to the solute cavity interfacial potential brings the force field predictions into much better agreement with each other and with implicit solvent (although some differences remain). We subsequently investigate ion pairing effects on the ferrocene and DMFc redox potentials within the [BMIM^+^][BF_4_^–^]-based organic electrolytes at systematically varying ion concentration. We find that ion interactions modulate the redox potentials in all cases, with the most substantial effects in the lower dielectric, DCE solvent, for which ∼5% ions by volume leads to redox potential shifts of ∼0.2 V relative to the bulk solvent, and the oxidation potential decreases by ∼0.8 V going from χ_IL_ = 0 to χ_IL_ = 1 ion content of the electrolyte. Linear response theory provides an insightful framework for understanding these oxidation potential shifts in terms of the anion coordination of the oxidized redox couple. Specifically, vertical ionization energies strongly depend on the anion coordination number, with the distribution of the latter being modulated by the ionic concentration.

## 2 Methods

Utilizing MD simulations, we compute the oxidation potentials of ferrocene (Fc) and DMFc in various solvent environments. The oxidation potentials are computed using a thermodynamic cycle that separates the oxidation process into electronic and solvation components. The electronic component, specifically the gas-phase, adiabatic ionization energy Δ*G*_(g)_^IP^, was taken from experimental values listed by NIST: 6.71 eV for Fc and 5.8 eV for DMFc.^54,55^ Our computations thus focus on the solvation component ΔΔ*G*_solv_^bulk^, which is the solvation free energy difference of the Fc/Fc^+^ and DMFc/DMFc^+^ redox couples in the ionic charge state compared to neutral charge state. To compute ΔΔ*G*_solv_^bulk^ within the solvent environments, we employ thermodynamic integration (TI) implemented via the Python interface of the OpenMM simulation package.^56^ Specific details regarding our implementation of TI are described in the Supporting Information. The MD simulations were performed for Fc/Fc^+^ oxidation within H_2_O, ACN, DCE, and TCM bulk solvents. Simulations were conducted utilizing both the OPLS-AA and SAPT-FF force fields, where OPLS-AA is a nonpolarizable force field developed by Jorgensen and Tirado-Rives,^48^ and SAPT-FF is an ab initio, polarizable force field,^16,50^ parametrized based on SAPT.^53,57^ For the simulations in water solvent, the TIP3P model^58^ was used in combination with OPLS-AA, and the SAPT-FF simulations employed a hybrid force field (as done previously^16^) with SWM4-NDP utilized for water–water interactions^59^ and specific SAPT-FF force field model used for water-Fc/Fc^+^ interactions. As benchmarks, densities and heats of vaporization were computed for each solvent using both the OPLS-AA and SAPT-FF models, as given in the Supporting Information. To model Fc/Fc^+^ within OPLS-AA simulations, a compatible Fc force field was used based on that developed by Lopes et al.^60^ but utilized as a flexible (not rigid) model. To model Fc/Fc^+^ within SAPT-FF simulations, new SAPT-FF parameters were fit for Fc (and DMFc) in this work, with parametrization details and benchmarks given in the Supporting Information. All necessary force field files utilized to conduct the simulations in the work are included in Supporting Information.

In addition to the bulk solvents, oxidation potentials of Fc/Fc^+^ and DMFc/DMFc^+^ were computed within organic electrolytes composed of the ionic liquid 1-butyl-3-methylimidazolium tetrafluoroborate ([BMIM^+^][BF_4_^–^]) mixed with either ACN or DCE solvent. The electrolyte systems systematically span the range of ionic compositions given in Table 1, ranging from pure solvent to pure ionic liquid. For these systems, simulations were conducted with the SAPT-FF force field only, with force field parameters for the [BMIM^+^][BF_4_^–^] ionic liquid taken from previous work.^16,52^ TI was utilized to compute the solvation free energy differences ΔΔ*G*_solv_^bulk^ of the Fc/Fc^+^ and DMFc/DMFc^+^ redox couples within these organic electrolytes. In addition, linear response theory (LR) was utilized to compute ΔΔ*G*_solv_^bulk^ for Fc/Fc^+^ within the [BMIM^+^][BF_4_^–^]/ACN and [BMIM^+^][BF_4_^–^]/DCE electrolytes; the LR predictions involve computation of ensemble average “vertical energy gaps” between the oxidized/neutral charge states of the redox couple, with specific details described in the Supporting Information.

**Table 1:** [BMIM^+^][BF_4_^–^]/ACN and [BMIM^+^][BF_4_^–^]/DCE Mixture Compositions Studied in This Work^a^

ion pairs	solvent	χ_IL_	Φ_IL_^ACN^	Φ_IL_^DCE^
0	1000	0.00	0.00	0.00
20	1000	0.02	0.07	0.05
50	1000	0.05	0.16	0.11
120	1000	0.11	0.31	0.23
220	750	0.23	0.53	0.42
220	400	0.35	0.68	0.58
220	200	0.52	0.81	0.73
300	200	0.60	0.86	0.78
300	75	0.80	0.94	0.91
400	0	1.00	1.00	1.00

a Listed is the number of ion pairs, solvent molecules, and corresponding [BMIM^+^][BF_4_^–^] mole fractions (χ_IL_) and volume fractions (Φ_IL_) of the different mixtures.

All MD simulations were performed with a single Fc (or DMFc) dissolved in each corresponding solvent/electrolyte. For all systems, initial configurations were created using the PackMol program.^61^ For nonpolarizable simulations, a Langevin thermostat was used to maintain the system at 300 K, using a 1 fs time step and a 1 ps^–1^ friction coefficient. For polarizable simulations, a dual-Langevin thermostat was used to maintain the nuclei at 300 K and Drude oscillators at 1 K, using a 1 fs time step, a 1 ps^–1^ friction coefficient, and 0.4 au masses applied to the Drude oscillators. Simulations of Fc with bulk solvents involved a single Fc in a box of 1000 solvent molecules. Solvent densities were equilibrated by performing 1 ns MD simulations in the *NPT* ensemble using a Monte Carlo barostat to maintain a pressure of 1 atm. For simulations involving ionic liquids, NPT equilibration time was increased to 10 ns. In all cases, equilibration was followed by 100 ns of production *NVT* simulation for each window of TI or each state in LR. The particle mesh Ewald method^62^ was used for long-range electrostatics, while van der Waals interactions were truncated at 1.4 nm.

Liquid/vapor interfacial potentials were additionally computed for each bulk solvent with both OPLS-AA and SAPT-FF force fields, as well as the [BMIM^+^][BF_4_^–^]/ACN and [BMIM^+^][BF_4_^–^]/DCE electrolytes with SAPT-FF force field. These potentials were computed by re-equilibrating the systems without Fc and extending the 3D periodic cell in the *z*-dimension to form a vacuum gap. The average charge density profile was then computed from the simulations, and the electrostatic potential profile was obtained from solution of the Poisson equation. For more details on the interfacial potential computations, see the Supporting Information.

## 3 Results and Discussion

### 3.1 Liquid–Vapor Interface Potentials: Force Field Dependence and Implications for Redox Potential Calculations

In electrochemistry, it is well appreciated that interface potentials give an important contribution to experimentally measured redox potentials.^11^ In experimental measurements, interface potentials are present at the working and reference electrode/electrolyte interfaces, as well as any solution-phase interface, such as liquid-junction potentials formed when a reference electrode is chosen with a different solvent (e.g., water) than the experimental system of interest (i.e., at working electrode). In computational redox potential prediction, interface potentials may or may not be explicitly considered.^1^ For example, for computation of the solvation contribution using an implicit solvent model, the solvent is treated as an “infinite” continuum dielectric medium with no explicit interfacial potential contribution. It would be tempting to conclude that such simplification would also apply to explicit solvent, MD predictions of solvation energies and redox potentials for bulk solvent environments as modeled with periodic boundary conditions (“infinite” solvent environment). However, we will show here that this is not the case, and an explicit consideration of interface potentials is critical when comparing redox potential predictions across different solvents and force fields.

**Figure 1:**
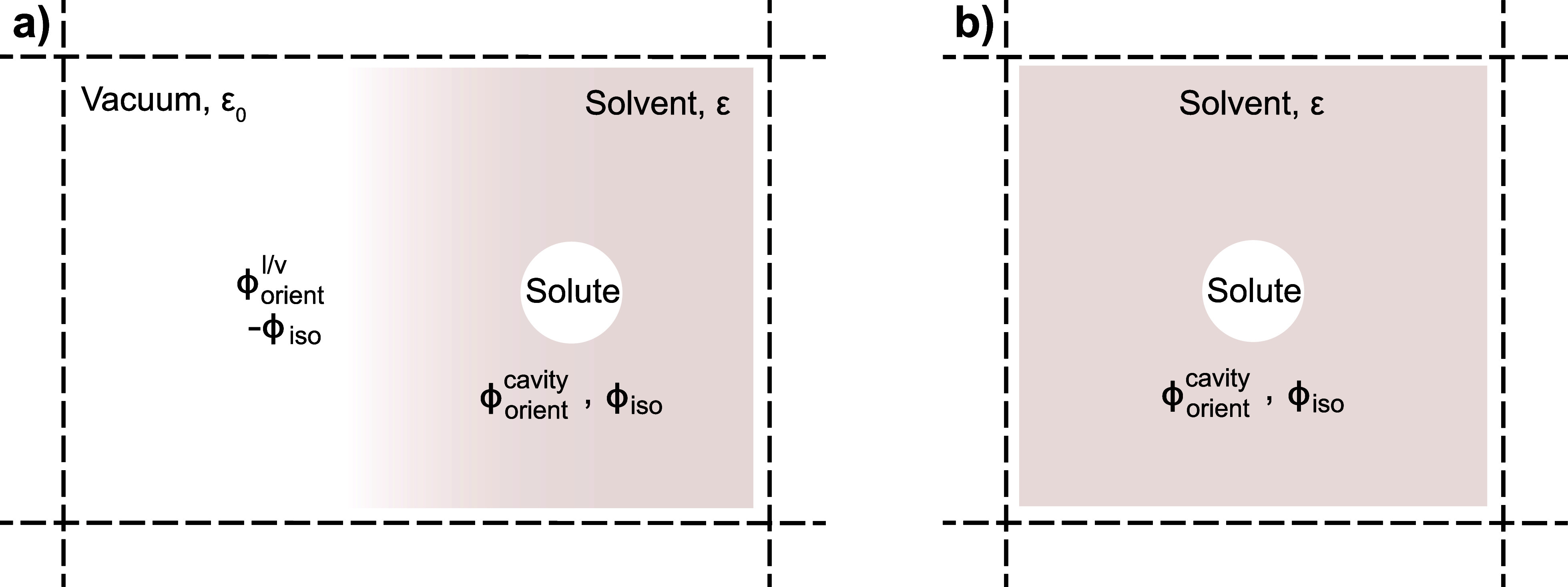
Depiction of possible simulation systems to define the absolute redox potential for a half reaction. In (a), the solvent/vacuum interface is explicitly considered, while in (b), the bulk solvent solution is “infinite” as modeled with periodic boundary conditions, so that no solvent/vacuum interface is explicitly present.

The importance of explicitly considering interface potential(s) is depicted schematically in Figure 1. In Figure 1, two different simulation systems are shown; in Figure 1a, the simulation system incorporates both solvent and vacuum, so that the liquid/vapor interface is explicitly considered (i.e., we use “vacuum” and “vapor” interchangeably). In contrast, Figure 1b depicts a redox solute solvated in an “infinite” bulk solvent environment, as modeled with periodic boundary conditions, so that there is no explicit consideration of the liquid/vapor interface. The “absolute” oxidation potential of a half reaction is commonly defined as the energy required to remove an electron off the redox moiety in solution and move the electron to vacuum.^63,64^ To move an electron into vacuum, the electron would have to cross the liquid/vapor interface, and thus, seemingly, Figure 1a presents the correct physical construct. The question then remains whether the “infinite” bulk solvent construct in Figure 1b (no liquid–vapor interface) would be an appropriate alternative reference system, given its natural use in MD simulations.

To explore this question, it is essential to note that an interfacial potential is also formed at the interface between the solvent and solute species, which we term the “cavity” potential, ϕ^cavity^ (in ref (7) this is termed “microscopic potential”, ϕ_micro_). This is because an interfacial potential results any time the symmetry of a charge distribution is broken by an interface, as rigorously defined by the equation^51^
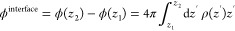
1where the interfacial potential ϕ^interface^ is the difference between electrostatic potential at different points in the system ϕ(*z*_2_), ϕ(*z*_1_) and depends on how the charge density ρ(*z*′) changes across the boundary (where the boundary is taken along the *z*-direction). Wilson et al. have shown that integration gives two contributions to the interfacial potential, namely, ϕ^interface^ = ϕ_orient_ + ϕ_iso_, where ϕ_orient_ comes from the orientation of molecular dipoles at the interface, and ϕ_iso_ is a quadrupolar contribution that does not depend on orientation (hence “isotropic”)
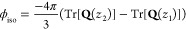
2where Tr[**Q**(*z*)] is the trace of the system quadrupole moment density within the phase at location “*z*”. For a liquid/vapor interface, ϕ_iso_ depends only on the quadrupolar density in the liquid as the density in the vapor is negligible. It is the contribution of ϕ_iso_ to the cavity potential (solute/solvent interface) that makes the simulation system in Figure 1b an unphysical reference for computing redox potentials in computer simulations. This is because any given force field utilized in a computer simulation produces an arbitrary/ill-defined value of ϕ_iso_, due to the fact that the molecular quadrupole moment trace is not a physically meaningful quantity in a force field. Note that for common atomic point charge-based force fields, electrostatic interactions can be formulated in terms of the multipole expansion of the molecular charge density (as defined by the atomic charges) and, in general, will have a contribution from the molecular quadrupole moment. From the Laplace condition, the molecular quadrupole trace does not contribute to electrostatic interactions between molecules, and thus different force fields may have different values for the quadrupole moment trace (e.g., subtract it altogether, “traceless” convention) without altering their description of intermolecular, electrostatic interactions.^65,66^ Furthermore very different values of ϕ_iso_ are predicted by DFT-based AIMD as compared to classical simulations employing force fields,^67−69^ and ϕ_iso_ is dramatically altered when atomic charge distributions are incorporated into the simulation analysis;^7,70^ consequently, all of these methods result in large discrepancy in predicted interfacial potentials.

Harder and Roux have previously presented an elegant discussion for the physical resolution of the ϕ_iso_ ambiguity.^7^ Because the value of ϕ_iso_ does not depend on the microscopic charge distribution ρ(*z*′) of the interface but only the bulk phase, quadrupole moment density (eq 2), both the liquid/vapor interface and the solute/solvent (cavity) interface will have equal and opposite values of ϕ_iso_ as predicted by any given force field or other method (e.g., DFT). Thus, for the system construct in Figure 1a, when an electron is removed from the solute redox moiety and transferred across the interface(s) to the vacuum phase, the contribution from interface potentials is
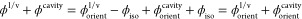
3

So that only the dipolar contributions to the interface potentials are present, and the quadrupolar contribution has canceled out. The necessity of considering both the liquid/vapor and solvent/solute (cavity) interfaces to cancel the ϕ_iso_ quadrupolar contribution has been recognized by other authors.^69^ Note that for the system construct in Figure 1b, the ϕ_iso_ term would contribute unphysically to any computed redox potential as the solute/solvent (cavity) interface is the only interface in the system.

**Figure 2:**
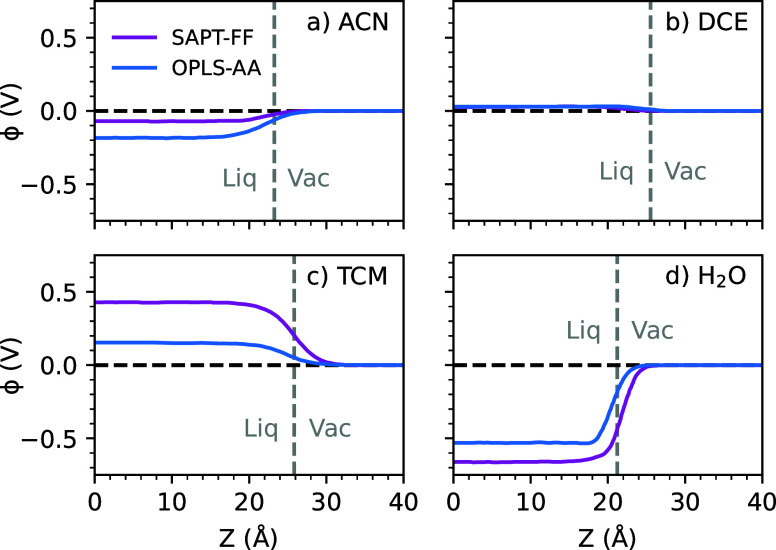
Electrostatic potentials, ϕ, computed across the liquid/vapor interface for bulk solvents (a) ACN, (b) DCE, (c) TCM, and (d) H_2_O for both nonpolarizable (OPLS-AA, blue) and polarizable (SAPT-FF, purple) force fields. Potentials are computed by solving the Poisson equation perpendicular to the liquid/vacuum interface (along the *z*-axis). The approximate location of the liquid-vacuum interface is given by a gray dashed line in each plot, with the liquid-phase to the left and vacuum-phase to the right of this line.

As an illustration of this concept, in Figure 2, we show liquid/vapor interfacial potential profiles as computed for ACN, DCE, H_2_O, and TCM from MD simulations, utilizing both the SAPT-FF and OPLS-AA force fields (details of the computations are given in Section 2). For the different solvent systems, magnitudes of the computed interfacial potentials range from smaller values of |ϕ^l/v^| ≤ 0.2 V for ACN and DCE solvents to larger 0.2 V  0.7 V values for H_2_O and TCM solvents, with predicted magnitudes depending on the force field. For reference, the value of ϕ^l/v^ for water, as computed from the TIP3P force field, agrees very well with literature values of ϕ^l/v^ ∼ −0.5 to −0.6 V, as computed with similar force fields.^7,69^ Contrary to intuition, this large value is not due to the dipolar contribution ϕ_orient_ but primarily results from the ϕ_iso_ contribution of the quadrupole term.^7,51^ The importance of the ϕ_iso_ contribution also explains the large predicted magnitude and force field dependence of ϕ^l/v^ for the TCM solvent (|ϕ^l/v^| = 0.43 eV for SAPT-FF and |ϕ^l/v^| = 0.15 eV for OPLS-AA force fields), even though the TCM molecule has a relatively small dipole moment. We will show that the often substantial contributions of ϕ_iso_ to the ϕ^l/v^ interface potentials will similarly lead to significant artifacts in predicted redox potentials from the system construct of Figure 1b and that a system construct similar to Figure 1a must be considered to cancel out the unphysical contribution of ϕ_iso_.

### 3.2 Redox Potential of Ferrocene in Bulk Solvents

We now discuss predictions for the absolute redox potential of ferrocene in the four different solvents, H_2_O, ACN, DCE, and TCM. We compare predictions from solvation free energies computed using polarizable SAPT-FF and nonpolarizable OPLS-AA force fields and present additional values computed with the COSMO implicit solvent model^71^ for comparison. The absolute oxidation potential is computed using a thermodynamic cycle and given as
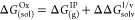
4where Δ*G*_(g)_^IP^ is the gas-phase ionization potential of ferrocene, and ΔΔ*G*_solv_^l/v^ is the difference in solvation free energy of the ferrocenium cation and the neutral ferrocene solute. In this work, Δ*G*_(g)_^IP^ = 6.71 eV is taken as the NIST recommended value for ferrocene.^54,55^ The superscript “l/v” indicates that the difference in solvation free energy should explicitly account for the contribution of the liquid/vapor interfacial potential, as in the system construct in Figure 1a. Practically, however, it is convenient to compute the solvation free energy difference from an “infinite” bulk solvent system (e.g., periodic boundary conditions) as in Figure 1b and then “add on” the liquid/vapor interfacial potential term as computed from separate simulations (e.g., Figure 2). Using this strategy, the solvation term is computed as
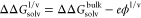
5

For which ΔΔ*G*_solv_^bulk^ is the difference in solvation free energy of the ion and neutral solute, computed from the bulk solvent system (no interface) in Figure 1b. As discussed in Section 2, ΔΔ*G*_solv_^bulk^ is computed using TI by incrementally scaling the charge state between the neutral ferrocene and ferrocenium cation. We make the commonly used approximation^32,33^ that van der Waals interactions are identical for the neutral and cation solute forms, so that all force field parameters are taken to be the same for ferrocene and ferrocenium, besides the charges. Also note that geometry differences are minor, with a structural relaxation energy of only 0.03 eV distinguishing the adiabatic compared to vertical ionization potential.^32^ We note a subtle difference in sign convention used in this work for defining ϕ^l/v^ compared to that in the literature. Because it is standard to report redox half potentials as reductions, ϕ^l/v^ is conventionally defined as ϕ^l^ – ϕ^v^, since the reducing electron is taken initially from a vacuum reference state and across the liquid/vapor interface (to reduce the solute).^7,69^ For H_2_O, this results in cited values of ϕ^l/v^ ∼ −0.5 to −0.6 V as computed with common water force fields.^7,69^ In this work, however, since we compute the oxidation potential of ferrocene, we take the opposite convention that ϕ^l/v^ = ϕ^v^ – ϕ^l^, corresponding to the oxidation process of removing the electron off ferrocene and across the liquid/vapor interface to the vacuum reference state. For water, as computed with the TIP3P force field, this gives a positive value of ϕ^l/v^ = 0.53 V (Figure 2) and thus shifts the oxidation potential to less positive values (eqs 4 and 5).

**Figure 3:**
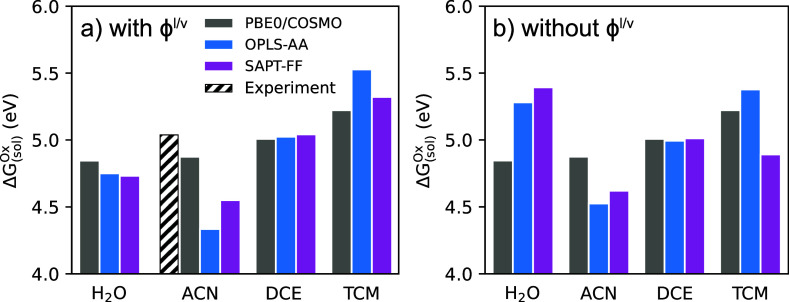
Comparison of absolute oxidation potentials (ΔG_(sol)_^Ox^) computed for Fc in 4 different bulk solvents: H_2_O, ACN, DCE, and TCM. The lanl2dz basis set was used for the PBE0/COSMO computation. OPLS-AA and SAPT-FF refer to the force fields used in TI computations. (a) Includes the interfacial potential correction (ϕ^l/v^); (b) does not include ϕ^l/v^. In panel (a), the experimental oxidation potential within ACN solvent is shown.

In Figure 3, we show computed values for the absolute oxidation potential of ferrocene in H_2_O, ACN, DCE, and TCM bulk solvents. Predictions from the SAPT-FF force field are shown in purple, predictions from the OPLS-AA force field are shown in blue, and predictions from PBE0/COSMO are shown in gray; in all cases, the denoted method is utilized to compute the solvation energy contribution, eq 5, with the gas-phase ionization potential taken as Δ*G*_(g)_^IP^ = 6.71 eV. To explicitly emphasize the contribution of the interface potential term ϕ^l/v^ to the oxidation potentials, we separately show predictions with (Figure 3a) and without (Figure 3b) the contribution of the ϕ^l/v^ interface term. Specifically, Figure 3a gives oxidation potentials computed with the full ΔΔ*G*_solv_^l/v^ solvation free energy term (eq 5), while Figure 3b gives oxidation potentials computed without the interface potential contribution, i.e., artificially setting ϕ^l/v^ = 0. Note that the interface potential applies to explicit solvent simulations and thus affects OPLS-AA and SAPT-FF predictions but is not relevant for implicit solvent, and thus PBE0/COSMO predictions are identical in Figure 3a,b. In Figure 3a, the experimental oxidation potential within ACN solvent is shown, as will be discussed subsequently.

An immediate takeaway is that oxidation potential predictions without the interfacial potential contribution (Figure 3b) show unphysical trends across the different solvents and seemingly large discrepancies between force fields (OPLS-AA vs SAPT-FF) and methods (explicit vs implicit solvent). For example, in Figure 3b, as predicted by SAPT-FF, the ferrocene oxidation potential is 0.4–0.5 eV higher in H_2_O than in the DCE and TCM solvents; this is contrary to intuition, as water has a much higher dielectric constant with expectedly larger (more negative) solvation energy of the ferrocenium cation, which would lead to lower oxidation potential. Also disturbing is the large discrepancy between explicit (OPLS-AA and SAPT-FF) and implicit (PBE0/COSMO) solvent predictions for the oxidation potential in H_2_O and the large discrepancy between OPLS-AA and SAPT-FF force field predictions for the oxidation potential in TCM. The 0.3–0.5 eV magnitude of these discrepancies (Figure 3b) is significantly greater than expected for solvation predictions of a large, “spherical” cation such as ferrocenium, for which one would expect closer agreement between methods. All of these anomalies stem from the fundamental necessity of including the interface potential contribution ϕ^l/v^ in order to remove the artificial ϕ_iso_ contribution from the cavity potential within the ΔΔ*G*_solv_^bulk^ values. As discussed in Section 3.1 and shown in Figure 2, ϕ_iso_ produces an often large (and arbitrary) contribution to the cavity potential and thus ΔΔ*G*_solv_^bulk^ values, which should be canceled by adding the interface potential contribution, as in eq 5. The conclusion is that trends in the oxidation potentials of Figure 3b are uninterpretable and unphysical, as they were computed without the ϕ^l/v^ interface contribution. Emphasis of this point is important, given that computation and discussion of interface potentials are sometimes neglected in computational predictions of redox potentials (or ion solvation free energies) with explicit solvent methods.

Figure 3a shows the computed ferrocene oxidation potentials with inclusion of the ϕ^l/v^ interface contribution; these values are thus physically meaningful, unlike those in Figure 3b. From the data in Figure 3a, it is evident that inclusion of the ϕ^l/v^ interface potential contribution corrects the anomalous (and unphysical) trends that were observed in Figure 3b. For example, all methods SAPT-FF, OPLS-AA, and PBE0/COSMO agree to within ∼0.1 eV for the oxidation potential prediction in bulk water (Figure 3a), which is a reasonable difference between methods. Furthermore, all methods predict larger oxidation potentials in the lower dielectric DCE and TCM solvents and smaller oxidation potentials in the higher dielectric H_2_O and ACN solvents (Figure 3a), an intuitive trend that was not observed when the interface contribution was omitted (Figure 3b). It is thus clear that inclusion of the interface potential contribution is crucial for qualitative, physical interpretation and not just for quantitative purposes; thus, unless otherwise stated, for the remainder of the paper, we will only present/discuss results computed with explicit evaluation/contribution of ϕ^l/v^.

We can now comment on the physical differences between the ferrocene oxidation potentials in different solvents, as predicted by the different methods in Figure 3a. First, we consider the implicit solvent predictions of the PBE0/COSMO method. Approximating ferrocenium as a spherical, monovalent ion, the Born model predicts a solvation energy difference of ∼20% for water (ε ∼ 78) compared to TCM (ε ∼ 5) solvents. A rough estimate using an ion radius of 3 Å (note that quantitative solute radii for use in Born model predictions require careful consideration^72^) would correspond to a ∼0.4–0.5 eV difference in ion solvation energy and thus ferrocene oxidation potential between water and TCM solvents, which is similar to the PBE0/COSMO predictions. Thus, the PBE0/COSMO predictions for ferrocene oxidation potential (Figure 3a) qualitatively follow the Born model, with decreasing oxidation potential (increasing ion solvation energy) with increasing solvent dielectric constant, TCM > DCE > ACN > H_2_O. In contrast, the differences between explicit solvent (OPLS-AA and SAPT-FF) and implicit solvent (PBE0/COSMO) predictions result from the atomistic liquid structure and correlations of the solvent surrounding the solute cavity and thus are more difficult to interpret. A powerful formalism for understanding how the solvation energy depends on underlying solvent liquid structure is given by linear response theory, in terms of the nonlocal, electrical susceptibility tensor of the solvent.^73^ For example, Dinpajooh and Matyushov have previously shown that the solute-size dependence of ion solvation energy in water significantly deviates from Born model predictions, as a result of the atomistic solvation structure and correlations.^73^ However, rigorous analysis would require computation of the nonlocal susceptibilities of the different TCM, DCE, ACN, and H_2_O solvents in the presence of a “ferrocene-like” solute cavity (and with the different OPLS-AA and SAPT-FF force fields), which is difficult without further approximation.^73^

In lieu of rigorous analysis,^73^ we simply speculate on the reasons for differences in the predictions of ferrocene oxidation potential by the explicit solvent (OPLS-AA and SAPT-FF) compared to implicit solvent (PBE0/COSMO) methods in Figure 3a. As the explicit and implicit solvent methods agree quite well (e.g., within ∼0.1 eV) for oxidation potential predictions with H_2_O and DCE solvents, discussion focuses on predicted oxidation potentials within ACN and TCM solvents, for which there are significant differences between methods. For the TCM solvent, the differences in predicted oxidation potentials likely originate primarily from OPLS-AA being a nonpolarizable and SAPT-FF being a polarizable force field. The oxidation potential of ferrocene within TCM solvent is predicted to be ∼0.2–0.3 V higher by OPLS-AA, as compared to either SAPT-FF (explicit solvent) or PBE0/COSMO (implicit solvent) methods (Figure 3a). This indicates that the ionic solvation energy in TCM is underpredicted by OPLS-AA (as compared with the other methods), in lieu of explicit treatment of electronic polarization. It is expected that nonpolarizable force fields with implicit description of electronic polarization will typically perform worse for describing ion solvation within low-dielectric compared to high-dielectric solvents, as the induced polarization within a low-dielectric bulk environment is much different than in the presence of ions.^50^ The other notable difference to mention is the oxidation potential of ferrocene in ACN solvent, for which OPLS-AA predicts an oxidation potential ∼0.22 V lower as compared to SAPT-FF, and predictions from both force fields are significantly lower as compared to implicit solvent predictions. In this regard, we note that SAPT-FF and OPLS-AA differ in their prediction of bulk ACN solvent properties more so than those for the other solvents (Table S1), which likely explains the differences in force field predictions for ferrocene oxidation potential in the ACN solvent.

We finally discuss comparisons of the predicted ferrocene oxidation potentials with experimental values. Figure 3a shows the experimental reference value for ferrocene oxidation potential within ACN, which is the solvent for which the majority of experimental values have been reported.^74^ While there exists substantial discrepancy in literature values,^32,74^ the recommended value is taken as ∼0.4 V vs SCE reference electrode.^74^ To compare with computational predictions, this must be converted to an “absolute scale”;^64^ we employ a conversion of 0.24 V between SCE and SHE reference^74^ and 4.4 V as the absolute potential of SHE.^1^ This gives an experimental value of 5.04 ± 0.1 V for the oxidation potential of ferrocene within ACN on an absolute scale, where the uncertainty has been estimated based on previous tabulation of numerous experimental data.^32^ Comparing to the predictions in Figure 3a, the implicit solvent prediction is closest to this experimental value, while both OPLS-AA and SAPT-FF force fields substantially underestimate the oxidation potential compared to the experimental value. We note that there is good agreement between implicit solvent prediction and experiment; the implicit solvent description may be justified given that bulk ACN is a homogeneous and aprotic solvent. The SAPT-FF force field contains the more rigorous physical description of interactions yet exhibits an error of ∼0.5 V in the oxidation potential prediction vs experiment (4.55 V vs 5.04 V). The source of discrepancy is quite unclear, considering that experimental gas-phase ionization potential of ferrocene was utilized so that the accuracy of our predicted oxidation potentials depends entirely on predicted solvation energies; it is not expected that the SAPT-FF force field would exhibit errors greater than ∼0.1 eV in predicted solvation energies (Supporting Information). The experimental values are, of course, complicated by the presence of liquid junction potentials introduced by the reference electrode, the values of which are unknown but may be on the order of ∼0.1 V.^75^ As noted in the Introduction, a recent work by Makos et al.^32^ reported very good agreement between first-principles prediction and experimental values for the oxidation potential of ferrocene in ACN; however, these authors did not consider interface potentials or the artificial ϕ_iso_ term (eq 3) inherent in the predictions.

### 3.3 Redox Potential of Ferrocene and DMFc in [BMIM^+^][BF_4_^–^]/ACN and [BMIM^+^][BF_4_^–^]/DCE Organic Electrolytes

We next discuss computations of the ferrocene oxidation potential in organic electrolytes composed of [BMIM^+^][BF_4_^–^] ionic liquid with either ACN or DCE solvent at systematically varying ion concentration. As discussed in the Introduction, the dependence of the oxidation potential on electrolyte composition has consequences for utilizing the Fc/Fc^+^ redox couple as a “universal reference electrode” standard. Additionally, ion pairing effects on redox potentials (which we will show are significant) are important to understand, as ion pairing will similarly affect reorganization energies dictating electron transfer kinetics,^28^ as well as reaction rates and selectivity in electrocatalysis processes.^76^ In addition to ferrocene, we also computed oxidation potentials of DMFc in the electrolyte systems. DMFc has also been proposed and investigated for use as a “universal reference” redox standard,^25^ and similar analysis of ion pairing effects on its oxidation potential is thus important for practical purposes. It is anticipated that qualitative trends in oxidation potentials will be analogous for ferrocene and DMFc but that quantitative solvent/electrolyte modulation of the oxidation potential will be less pronounced for DMFc since it is a larger molecule/ion.

**Figure 4:**
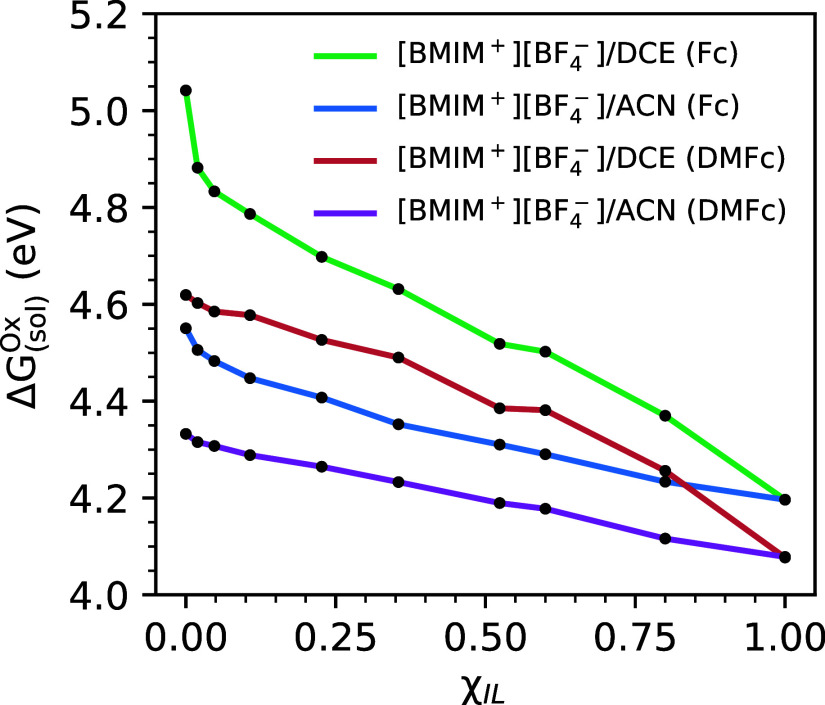
Absolute oxidation potentials (Δ*G*_(sol)_^Ox^) of Fc and DMFc computed in [BMIM^+^][BF_4_^–^]/ACN and [BMIM^+^][BF_4_^–^]/DCE organic electrolytes of varying ionic liquid mole fraction, χ_IL_.

In Figure 4, we show computed absolute oxidation potentials for Fc and DMFc in the [BMIM^+^][BF_4_^–^] ionic liquid-based electrolytes with either ACN or DCE solvent at varying ion concentration. The specific ion concentrations studied are given in Table 1, in terms of the IL mole fraction χ_IL_ (Table 1 also gives the corresponding ion volume fraction of the electrolytes). Absolute oxidation potentials were computed according to eq 5, thus including the contribution of ϕ^l/v^, with the gas-phase ionization potential for ferrocene taken as Δ*G*_(g)_^IP^ = 6.71 eV and for DMFc taken as Δ*G*_(g)_^IP^ = 5.8 eV, based on experimental values.^54,55^ We note that all results presented in this Section and in the remainder of the work are obtained from simulations utilizing the polarizable (ab initio), SAPT-FF force field. From the data in Figure 4, it is clear that oxidation potentials of both ferrocene and DMFc vary as a function of the solvent and ion content of the electrolyte. The most substantial variation is observed for ferrocene within DCE-based electrolytes, for which the oxidation potential shifts from larger value of ∼5.05 V to smaller value of ∼4.2 V for increasing ion concentration toward the pure [BMIM^+^][BF_4_^–^] ionic liquid limit. For the same series of [BMIM^+^][BF_4_^–^]/DCE electrolytes, the oxidation potential of DMFc varies from ∼4.6 V to ∼4.1 V going from the bulk solvent to pure ionic liquid; this variation is significant, but, as expected, less pronounced compared to that for the smaller ferrocene redox couple. The trends in oxidation potentials within the [BMIM^+^][BF_4_^–^]/ACN electrolytes are qualitatively similar in that oxidation potentials decrease with increasing ion content, but the variation is not as pronounced as in the DCE-based electrolytes, due to the fact that solvation energies within the bulk ACN solvent are more similar to those in the pure [BMIM^+^][BF_4_^–^] ionic liquid. Our predictions are in qualitative agreement with an experimental study of Bao et al.,^13^ in which it was found that the oxidation potential of ferrocene decreased by ∼0.6 V with increasing ion content in a relative low dielectric, dichloromethane solvent (note that dichloromethane with ε ∼ 9 and DCE with ε ∼ 10 have similar dielectric strength). We note, however, that this experimental study examined a lower ionic concentration range than has been considered here.

To rationalize the oxidation potential trends shown in Figure 4, we first focus on the limiting behavior in the bulk ACN and DCE solvents and pure [BMIM^+^][BF_4_^–^] ionic liquid. As discussed in Section 3.2, the higher oxidation potentials of ferrocene and DMFc in DCE compared to ACN can be rationalized by the lower dielectric constant of DCE and anticipated lower magnitude solvation energy of the oxidized species within this solvent, although the quantitative differences are larger than what would be predicted by the Born model. However, differences in oxidation potentials within the bulk ACN/DCE solvents compared to [BMIM^+^][BF_4_^–^] ionic liquid cannot be rationalized by solvent dielectric constants or “continuum” description of the ionic solvation energy. For example, based on the dielectric constants of ε ∼ 12–14 for [BMIM^+^][BF_4_^–^]^77−79^ and ε ∼ 38 for ACN, the Born model would predict a larger magnitude ionic solvation energy and thus lower oxidation potential for ferrocene within ACN compared to [BMIM^+^][BF_4_^–^], which is contrary to the predicted trend in Figure 4. Such qualitative deviations from Born model predictions must consequently be rationalized by atomistic length scale structure and correlations of the different solvent systems.^73^

In a recent study, Renfro et al. provided a qualitative explanation for differences in solvation energy of model solute ions within [BMIM^+^][BF_4_^–^] ionic liquid compared to bulk ACN solvent.^18^ Based on linear response theory and subsequently invoking a “homogeneous susceptibility” approximation, the solvation energy of a (spherical) ion is given in terms of the longitudinal electrical susceptibility of the solvent. The oscillatory ion structure of the [BMIM^+^][BF_4_^–^] ionic liquid results in a pronounced peak in the electrical susceptibility at *k* ∼ 1 Å^–1^ reciprocal space length scales, which leads to an enhancement in ionic solvation energy for specific size ions.^17,18^ This “overscreening effect” is thus the origin for larger magnitude solvation energies (and hence lower oxidation potentials) within the [BMIM^+^][BF_4_^–^] ionic liquid as compared to the bulk ACN or DCE solvents and must fundamentally be interpreted from an atomistic description of the ionic solvation process.^18^

**Figure 5:**
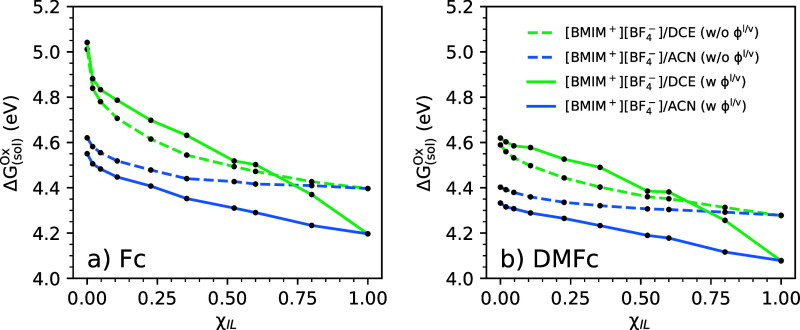
Absolute oxidation potentials (Δ*G*_(sol)_^Ox^) of (a) Fc and (b) DMFc computed in [BMIM^+^][BF_4_^–^]/ACN and [BMIM^+^][BF_4_^–^]/DCE organic electrolytes. Data labeled “w ϕ^l/v^” are identical to data in Figure 4 and include the computed interface potential ϕ^l/v^. Data labeled “w/o ϕ^l/v^” are computed by artificially setting ϕ^l/v^ = 0 in eq 5.

**Figure 6:**
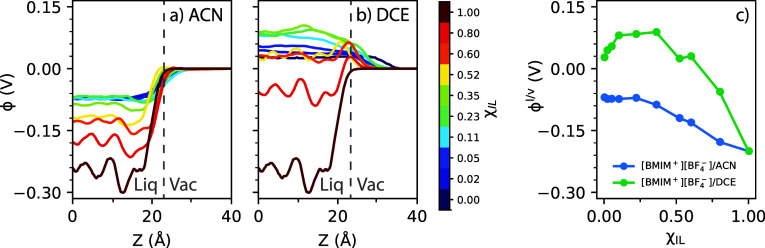
Electrostatic potentials, ϕ, computed across the liquid/vapor interface for (a) [BMIM^+^][BF_4_^–^]/ACN and (b) [BMIM^+^][BF_4_^–^]/DCE organic electrolytes at varying ion content, χ_IL_. The approximate location of the liquid-vacuum interface is given by a gray dashed line in each plot, with the liquid-phase to the left and vacuum-phase to the right of this line. (c) Interfacial potentials, ϕ^l/v^, as a function of ion content, χ_IL_, computed for each potential profile given in (a,b).

We next turn to discussion of the dependence of Fc and DMFc oxidation potentials on ionic concentration within the [BMIM^+^][BF_4_^–^]/ACN and [BMIM^+^][BF_4_^–^]/DCE organic electrolytes. First, it is important to note that computed oxidation potentials in Figure 4 seemingly exhibit statistical uncertainty, particularly within the [BMIM^+^][BF_4_^–^]/DCE electrolytes, as a smooth trend line does not fit the data points at varying ionic concentration, χ_IL_. This uncertainty in predicted oxidation potentials is due to computation of the interfacial potential, ϕ^l/v^, and not computation of ΔΔ*G*_solv_^bulk^ in evaluation of eq 5. To illustrate this, in Figure 5, we show corresponding oxidation potentials of Fc and DMFc computed with or without inclusion of the interfacial potential; explicitly, the oxidation potentials labeled “w/o ϕ^l/v^” are computed by artificially setting ϕ^l/v^ = 0 in eq 5. From Figure 5, it is clear that the oxidation potentials labeled “w/o ϕ^l/v^” show a smooth trend with increasing ion content, χ_IL_, of the organic electrolytes. Evidently then, the associated uncertainty in oxidation potentials computed with inclusion of the interface potential (“w ϕ^l/v^”) comes from computation of the interface potential itself. In Figure 6, we show computed electrostatic potential profiles across the liquid/vapor interface and corresponding derived interfacial potentials ϕ^l/v^ for the [BMIM^+^][BF_4_^–^]/ACN and [BMIM^+^][BF_4_^–^]/DCE organic electrolytes. While computed interfacial potentials ϕ^l/v^ for the [BMIM^+^][BF_4_^–^]/ACN electrolytes show a reasonably smooth and monotonic dependence on ion concentration χ_IL_, this is not the case for the [BMIM^+^][BF_4_^–^]/DCE organic electrolytes. For the [BMIM^+^][BF_4_^–^]/DCE electrolytes, ϕ^l/v^ shows a nonmonotonic trend with χ_IL_, and greater statistical uncertainty, likely a result of ion pairing and clustering that is observed to occur in [BMIM^+^][BF_4_^–^]/DCE electrolytes, due to the lower dielectric constant of the DCE solvent.^16^ Ion pairing and clustering in [BMIM^+^][BF_4_^–^]/DCE electrolytes lead to statistical uncertainty when sampling liquid/vapor interfacial profiles, thus creating uncertainty in the prediction of ϕ^l/v^ (Figure 6) and corresponding oxidation potentials (Figure 4) within these systems.

**Figure 7:**
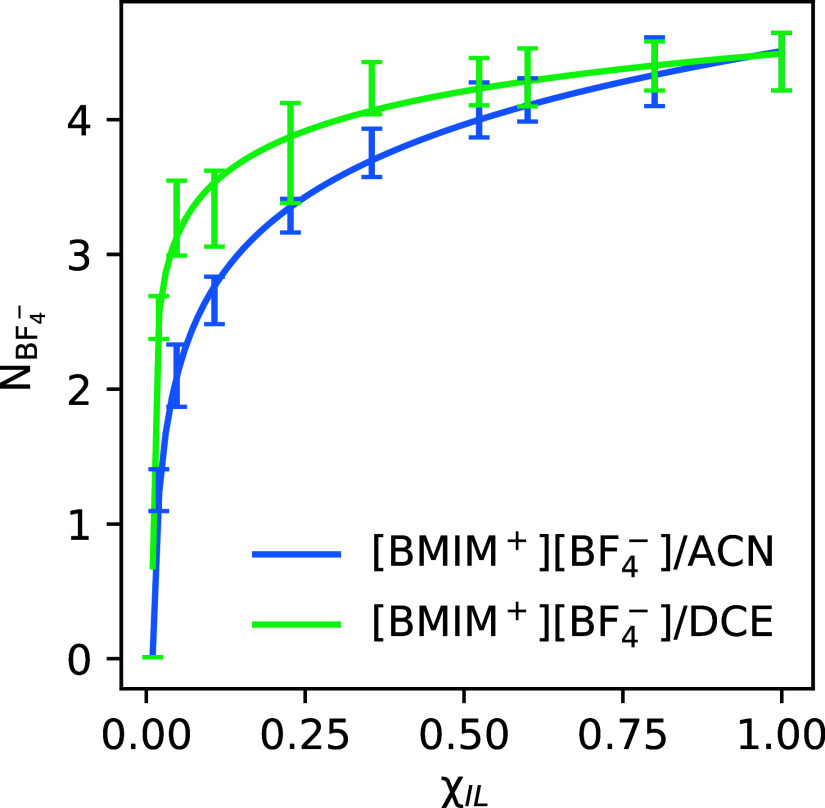
Plot comparing coordination number of BF_4_^–^ (*N*_BF4_^–^) around Fc^+^ within varying mole fractions [BMIM^+^][BF_4_^–^] (χ_IL_) for both ACN and DCE solvents. Coordination number was computed using a hard cutoff of 6.5 Å from the iron center of Fc^+^.

The oxidation potentials of Fc and DMFc computed without the interfacial potential contribution (“w/o ϕ^l/v^”, Figure 5) were shown solely to illustrate the associated uncertainty and are not physically interpretable given the discussion in Sections 3.1 and 3.2. Henceforth, we focus on the physical interpretability of the predictions in Figure 4, as computed with inclusion of ϕ^l/v^. The interesting physical effect left to discuss is the dependence of the Fc and DMFc oxidation potentials on the ion content χ_IL_ of the [BMIM^+^][BF_4_^–^]/ACN and [BMIM^+^][BF_4_^–^]/DCE electrolytes. The likely cause/explanation for this dependence is ion pairing/coordination of the Fc^+^/DMFc^+^ cation with surrounding ions in the electrolyte, which would modulate the solvation energy of the oxidized species and, thus, the overall oxidation potential. As a metric of the ionic environment surrounding the Fc^+^ cation, we compute the average coordination number ” of BF_4_^–^ anions surrounding the Fc^+^ cation in the different [BMIM^+^][BF_4_^–^]/ACN and [BMIM^+^][BF_4_^–^]/DCE electrolytes as a function of ion content, χ_IL_. These computed  coordination numbers are shown in Figure 7. As expected, the  anion coordination of the Fc^+^ cation increases monotonically with increasing ion content of the electrolyte. For specific ion concentrations approaching the ionic liquid limit,  anion coordination is higher in the [BMIM^+^][BF_4_^–^]/DCE compared to [BMIM^+^][BF_4_^–^]/ACN electrolyte, due to the lower dielectric constant and thus weaker screening of the DCE solvent. In fact in DCE-based electrolytes at low to moderate ion concentrations, ions exist primarily as ion pairs or clusters due to weak screening.^16^ The trends in coordination number in Figure 7 are qualitatively very similar to those computed between BMIM^+^ cations and BF_4_^–^ anions in the [BMIM^+^][BF_4_^–^]/ACN and [BMIM^+^][BF_4_^–^]/DCE electrolytes (see Figure 5 in ref (16)), suggesting the trends are not specific to the nature of the Fc^+^ cation. While the trends in Figure 7 appear markedly nonlinear, previous work has shown that analogous trends appear much more linear when coordination number is plotted against ion volume fraction instead of ion mole fraction of the electrolyte.^16^

From comparison of Figures 4 and 7, it is clear that the anion coordination  of Fc^+^ correlates with the oxidation potential of ferrocene in the different concentration electrolytes; higher anion coordination correlates with greater magnitude solvation energy of the oxidized species, resulting in lower oxidation potential. A direct quantification of how the anion coordination modulates the oxidation potential is possible through the application of linear response theory. From linear response theory, the difference in solvation free energy between the neutral and oxidized state, ΔΔ*G*_solv_^bulk^, can be expressed in terms of computed “vertical energy gaps”^80^
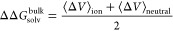
6where Δ*V* = *V*_ion_ – *V*_neutral_ is the difference in system energy when the redox couple is in the ionic (Fc^+^) or neutral (Fc) oxidation state, and ⟨...⟩_ion/neutral_ denotes an ensemble average defined by the Hamiltonian corresponding to the ion/neutral state. This linear response formula is advantageous for quantifying how the specific ionic solvation environment modulates the solvation contribution ΔΔ*G*_solv_^bulk^ to the oxidation potential because the ensemble averages ⟨Δ*V*⟩_ion_ and ⟨Δ*V*⟩_neutral_ can be dissected in terms of different coordination environments. In other words, the average of ⟨Δ*V*⟩_ion/neutral_ can be computed from snapshots in the simulation with a given anion coordination number , and the ensemble average reformulated as
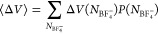
7where  is the probability distribution for a given coordination number , and  is the average of Δ*V* taken over simulation snapshots with coordination number .

**Figure 8:**
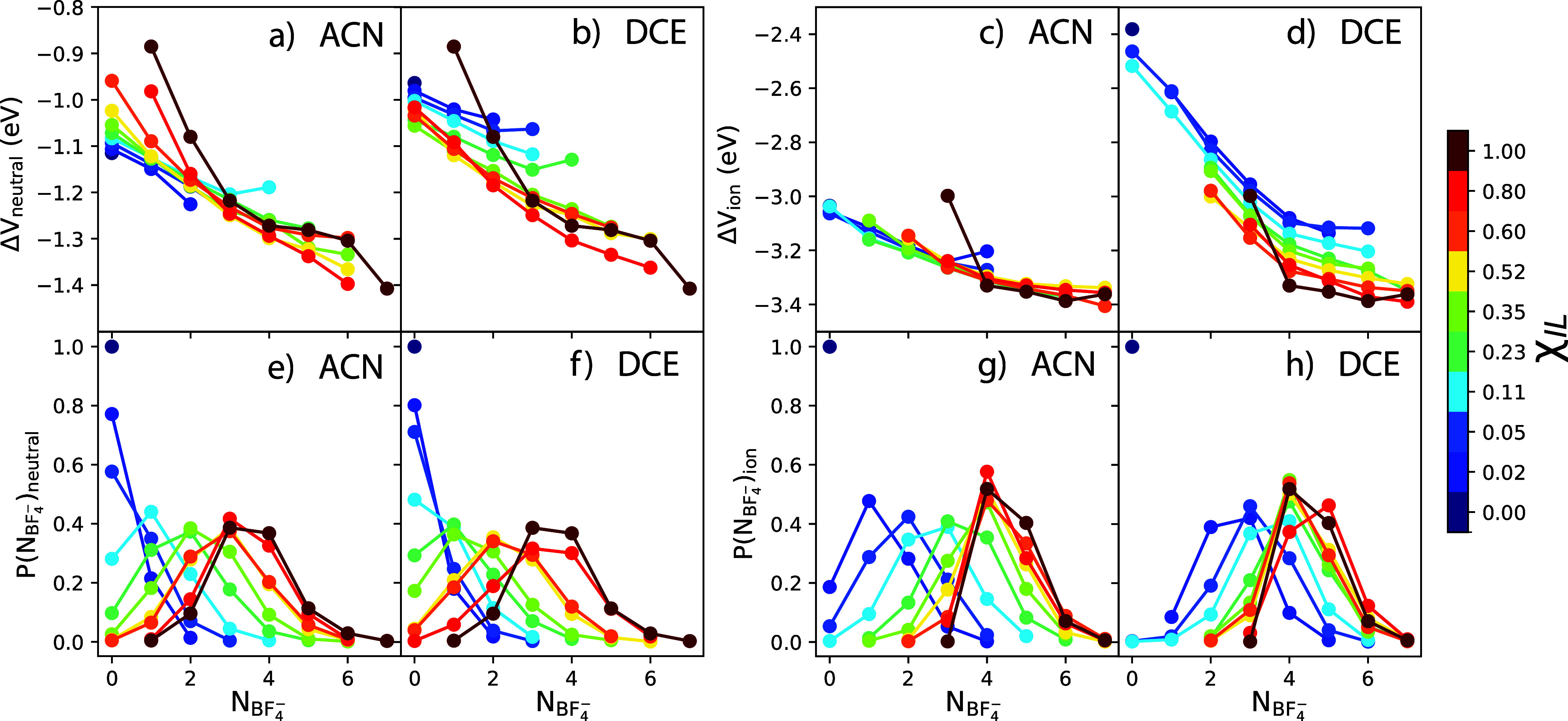
Vertical energy gaps computed for Fc in [BMIM^+^][BF_4_^–^]/ACN and [BMIM^+^][BF_4_^–^]/DCE mixtures of varying [BMIM^+^][BF_4_^–^] mole fractions (χ_IL_), averaged over specific coordination numbers of . Distributions of Δ*V*_neutral_ versus  for [BMIM^+^][BF_4_^–^]/ACN and [BMIM^+^][BF_4_^–^]/DCE mixtures are shown in (a,b). Similar distributions of Δ*V*_ion_ versus  for [BMIM^+^][BF_4_^–^]/ACN and [BMIM^+^][BF_4_^–^]/DCE mixtures are shown in (c,d). Probability distributions of anion coordination, , for (a–d) are given in (e–h), respectively.

Figure 8 shows computed vertical energy gaps for Fc/Fc^+^ as a function of anion coordination number , and corresponding probability distribution , within the different electrolytes. In Figure 8a,b, Δ*V*_neutral_ is shown for the [BMIM^+^][BF_4_^–^]/ACN and [BMIM^+^][BF_4_^–^]/DCE electrolytes, respectively. Because Δ*V*_neutral_ is computed from a trajectory sampled with ferrocene in the neutral charge state (Fc), the ionic environment is not organized for optimal solvation of the Fc^+^ ion. At lower ionic concentrations (e.g., χ_IL_ ≤ 0.1), it is thus improbable/unlikely to have substantial anion coordination of Fc/Fc^+^, and values of  are most probable (Figure 8e,f). In this low ionic regime, more negative values of Δ*V*_neutral_ are observed for the ACN compared to DCE electrolytes, since in this case, the dielectric constant of the solvent is the major determiner of ion solvation. Note that the probability distributions  for the neutral Fc charge state (Figure 8e,f) are quite similar for the [BMIM^+^][BF_4_^–^]/ACN and [BMIM^+^][BF_4_^–^]/DCE electrolytes, given there is no strong interaction to bias the electrolyte ion distribution around the neutral solute species.

In the pure [BMIM^+^][BF_4_^–^] ionic liquid limit, the Δ*V*_neutral_ dependence on anion coordination number  (Figure 8a,b) appears to exhibit two different trends at regimes of low  and high  anion coordination to Fc/Fc^+^. In the pure ionic liquid, since there are no neutral solvent molecules, a low anion coordination environment  implies that Fc/Fc^+^ must be surrounded by a number of BMIM^+^ cations. While ferrocene may be solvated by either the nonpolar alkyl chain or positively charged imidazolium ring of the BMIM^+^ cation, in either case, this is a suboptimal environment for the oxidation to Fc^+^, and hence there is a much more positive value of Δ*V*_neutral_ in the case of more surrounding cations (e.g., at low anion  coordination). The value of Δ*V*_neutral_ then rapidly decreases to more negative values as the solvation environment for Fc^+^ is substantially optimized with increasing anion coordination from  to . For ferrocene coordination of  within the [BMIM^+^][BF_4_^–^] ionic liquid, Δ*V*_neutral_ varies only marginally, suggesting similar electrostatic solvation environments. Finally, at the highest anion coordination of  within the ionic liquid, Δ*V*_neutral_ drops sharply by >0.1 eV, indicating a more favorable solvation environment for Fc^+^.

In Figure 8c,d, the Δ*V*_ion_ contribution is shown for the [BMIM^+^][BF_4_^–^]/ACN and [BMIM^+^][BF_4_^–^]/DCE electrolytes, respectively. The difference between Δ*V*_ion_ and previously discussed Δ*V*_neutral_ is that Δ*V*_ion_ is computed from simulation trajectories sampled with ferrocene in its oxidized charge state (Fc^+^). Thus, Δ*V*_ion_ values are much larger in magnitude (more negative) compared to Δ*V*_neutral_ values, since the solvation environment is optimized for the Fc^+^ cation. The dependence of the Fc/Fc^+^ redox potential on electrolyte composition (through the ΔΔ*G*_solv_^bulk^ term, eq 6) is thus primarily due to solvent modulation of Δ*V*_ion_ because of its much larger magnitude compared to Δ*V*_neutral_. Comparison of Figure 8c with Figure 8d provides explanation for why the oxidation potential of ferrocene (Figure 4) is much more substantially modulated by ion content χ_IL_ in [BMIM^+^][BF_4_^–^]/DCE compared to [BMIM^+^][BF_4_^–^]/ACN electrolytes. In the [BMIM^+^][BF_4_^–^]/DCE electrolytes, Δ*V*_ion_ markedly decreases to more negative values with increasing anion coordination from  to . The magnitude of this effect is substantial, with Δ*V*_ion_ being ∼0.6 eV more negative for anion coordination  compared to , as evaluated for electrolyte compositions of χ_IL_ = 0.02–0.11. The dependence of Δ*V*_ion_ on the anion coordination number  is particularly pronounced in the [BMIM^+^][BF_4_^–^]/DCE electrolytes because of the low dielectric constant and relatively poor screening of the DCE solvent. Ion–ion interactions are stronger in DCE compared to ACN solvent,^50^ and thus ion pairing/coordination has a markedly larger impact on the solvation energy of Fc^+^ (and thus oxidation potential of ferrocene) within the DCE-based electrolytes.

It is interesting to note that for the [BMIM^+^][BF_4_^–^]/ACN electrolytes, the dependence of Δ*V*_ion_ on the anion coordination number  largely collapses to a single curve for widely varying ion content from dilute χ_IL_ = 0.02 to the pure ionic liquid limit χ_IL_ = 1.0 (Figure 8c). This implies that the major effect of varying ion concentration in these electrolytes is to change the distribution or probability  of anion coordination for the Fc/Fc^+^ redox couple, as shown in Figure 8g, which correspondingly modulates the oxidation potential through eq 7. This collapse of Δ*V*_ion_ vs  to a single curve for all compositions χ_IL_ of [BMIM^+^][BF_4_^–^]/ACN electrolytes is unique and not observed for the [BMIM^+^][BF_4_^–^]/DCE electrolyte systems. For the [BMIM^+^][BF_4_^–^]/DCE electrolytes, each electrolyte composition χ_IL_ exhibits a qualitatively similar Δ*V*_ion_ vs  curve/trend but with curves systematically shifted to more negative Δ*V*_ion_ values at increasing ion content (greater χ_IL_). Collapse to a single Δ*V*_ion_ vs  curve for different ion composition [BMIM^+^][BF_4_^–^]/ACN electrolytes implies that pure ACN solvent and pure [BMIM^+^][BF_4_^–^] ionic liquid provide relatively similar solvation environments beyond the local ion coordination shell, such that the local coordination  is the primary metric dictating ionic composition effects. We note that in both Figure 8c,d, there is seemingly an outlier point for Δ*V*_ion_ at  as computed for the pure [BMIM^+^][BF_4_^–^] ionic liquid (χ_IL_ = 1.0). This is simply because  is a very rare/improbable coordination environment for Fc^+^ within the [BMIM^+^][BF_4_^–^] ionic liquid, as seen in the anion coordination distributions in Figure 8g,h; this Δ*V*_ion_ value thus contributes negligibly to the ΔΔ*G*_solv_^bulk^ solvation contribution within the ionic liquid system.

**Figure 9:**
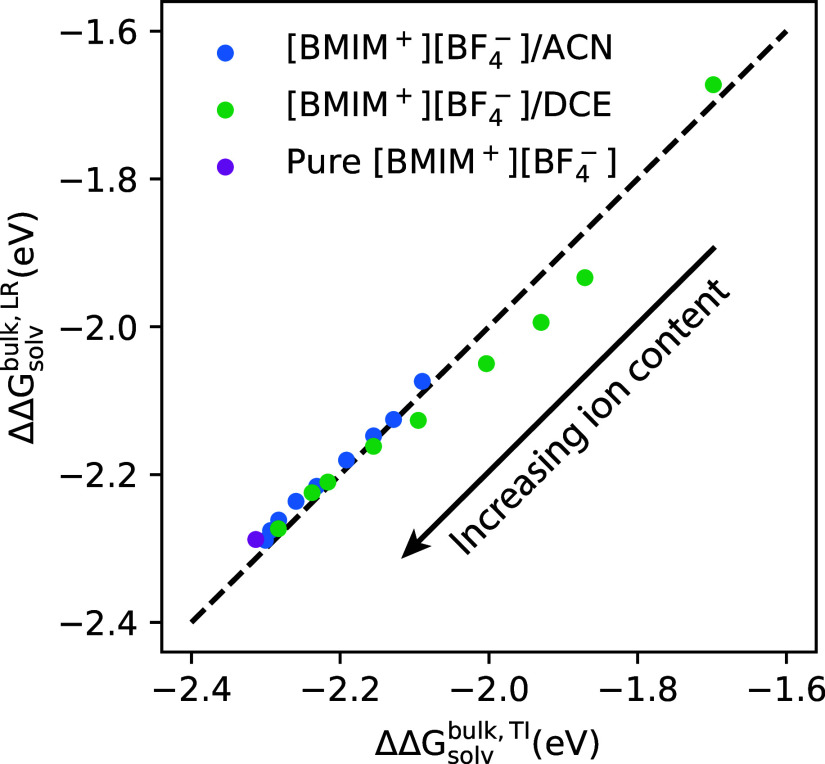
Comparison of ΔΔ*G*_solv_^bulk^ computed using TI (*x*-axis) and linear response theory (LR, *y*-axis) for [BMIM^+^][BF_4_^–^]/ACN and [BMIM^+^][BF_4_^–^]/DCE electrolytes of varying composition. The arrow indicates that the higher ionic concentration systems correspond to data points on the lower left of the graph.

As illustrated by the data presented in Figure 8, the linear response formalism (eq 6) provides an insightful framework for interpreting how ion pairing and correlation modulate oxidation potentials within concentrated organic electrolytes. However, one might question the accuracy of the linear response approximation for these organic electrolyte systems, particularly over the wide ionic composition range spanning from χ_IL_ = 0.0 to 1.0. To test the linear response approximation, eq 6 can be used to predict ΔΔ*G*_solv_^bulk^ values for Fc/Fc^+^ within the different electrolytes and compared to the ΔΔ*G*_solv_^bulk^ values predicted from TI that were utilized in the computed redox potentials shown in Figure 4. This comparison of ΔΔ*G*_solv_^bulk^ predictions from both linear response theory “ΔΔ*G*_solv_^bulk,LR^” and TI “ΔΔ*G*_solv_^bulk,TI^” is given in Figure 9, for the [BMIM^+^][BF_4_^–^]/ACN and [BMIM^+^][BF_4_^–^]/DCE electrolytes at varying ion concentrations. For most of the systems including the pure [BMIM^+^][BF_4_^–^] ionic liquid, bulk ACN and DCE solvents, and [BMIM^+^][BF_4_^–^]/ACN electrolytes at all ion compositions, the linear response approximation is quite good, predicting ΔΔ*G*_solv_^bulk^ values in close quantitative agreement with TI predictions. This conclusion is consistent with previous work that found linear response to be a good approximation for redox processes in ionic liquids and organic solvents such as ACN.^18,81^ However, it is observed in Figure 9 that data for four electrolytes are “outliers” in that the linear response predictions exhibit systematic error of ∼0.05–0.1 eV; these four systems are the [BMIM^+^][BF_4_^–^]/DCE electrolytes at χ_IL_ = 0.02, 0.05, 0.11, and 0.23 ion compositions. Previously, it has been shown that [BMIM^+^][BF_4_^–^]/DCE electrolytes of low-to-moderate ion composition exhibit substantial ion correlation, in the form of ion pairing and clustering,^16^ caused by the low dielectric constant and relatively poor screening of the DCE solvent. The substantial ion pairing/clustering in the χ_IL_ = 0.02–0.23 [BMIM^+^][BF_4_^–^]/DCE electrolytes is likely the origin for the quantitative errors in the linear response predictions for these systems. Despite these errors, it is overall concluded that the linear response formalism (eq 6) generally provides semiquantitative accuracy while serving as an insightful framework for interpreting how ion pairing/correlation within electrolytes modulates the oxidation potential of Fc/Fc^+^ (Figure 8).

## 4 Conclusions

We have presented a comprehensive MD study of solvent and ion modulation of the oxidation potentials of ferrocene and DMFc in organic solvents and ionic liquid-based organic electrolytes. While historically the majority of redox potential predictions in the literature have employed implicit solvent models, explicit solvent MD predictions of solvation energies are an important alternative for improved accuracy and capturing effects such as ion pairing/correlation that have been suggested as substantially modulating redox potentials in organic electrolytes. In this regard, we have provided a detailed numerical analysis of the importance of interfacial potentials ϕ^l/v^ in explicit solvent computations of ionic solvation energies required for redox potential predictions. As we have discussed, ϕ^l/v^ must be explicitly computed from simulations of the liquid/electrolyte of interest utilizing the specific employed force field and incorporated into the solvation energy prediction; otherwise, the ϕ_iso_ term from the solute cavity leads to unbounded and artificial errors from the force field quadrupole trace that can be as substantial as ∼0.5 eV. The conclusion is that an infinite, bulk solvent system as modeled with periodic boundary conditions is not an appropriate reference system for defining an absolute redox potential, and the simplest physically valid reference system (with explicit solvent) must exhibit a solution phase interface, such as liquid/vapor interface, as depicted in Figure 1a.

Our predictions of ferrocene and DMFc oxidation potentials in [BMIM^+^][BF_4_^–^]/ACN and [BMIM^+^][BF_4_^–^]/DCE electrolytes demonstrate that these oxidation potentials significantly decrease to less positive values with increasing ion content of the electrolyte. The magnitude of the decrease depends on both the solute and nature of the electrolyte. The extreme case is ferrocene in [BMIM^+^][BF_4_^–^]/DCE electrolytes, for which the oxidation potential decreases by ∼0.8 V going from χ_IL_ = 0 to χ_IL_ = 1 ion content of the electrolyte! Changes in oxidation potential are less pronounced (but still significant) in [BMIM^+^][BF_4_^–^]/ACN electrolytes and less pronounced for the larger DMFc solute. However, there is still a ∼0.2 V decrease in the DMFc oxidation potential with increasing ion content from χ_IL_ = 0 to χ_IL_ = 1 within [BMIM^+^][BF_4_^–^]/ACN electrolytes. While substantial dependence of the ferrocene oxidation potential on electrolyte ion concentration has been previously observed experimentally,^13^ our simulations provide additional physical insight as to the origin of this behavior in terms of ion pairing effects on the solvation energy. Employing linear response theory, the ion concentration modulation of solvation energies is formulated in terms of distributions of anion coordination of the redox couple and vertical energy gaps evaluated for specific anion coordination environments.

The results of our study have practical importance while raising additional questions as to interpretation of experimental redox potential data. Clearly, the “Strehlow assumption” that the redox potential of ferrocene (or even DMFc) is solvent/electrolyte independent^12^ is not quantitatively valid due to both solvent and ion modulation of the ferrocenium solvation energy. However, the accurate computational prediction of ferrocene/DMFc redox potentials in different organic electrolytes (as in Figure 4) provides the required “solvation correction” to normalize different electrochemistry measurements that are referenced to the Fc/Fc^+^ or DMFc/DMFc^+^ redox couple. The puzzling question remaining from our study is why our predicted oxidation potentials of ferrocene in bulk solvents exhibit sometimes large (e.g., ∼ 0.5 V) deviations from corresponding experimental oxidation potentials? Because we have utilized the experimental gas-phase ionization potential of ferrocene, the accuracy of our predicted oxidation potentials depends entirely on predicted solvation energies. For solvation energy predictions, we have utilized explicitly polarizable, ab initio force fields parametrized to high level electronic structure theory (DFT-SAPT^53^), and it seems unlikely that these predictions would be in error by order ∼0.5 eV. Thus, despite recent publications highlighting excellent agreement between first-principles redox potential predictions with experimental values,^32^ we believe that accurate first-principles prediction of redox potentials within organic electrolytes is still an ongoing challenge. Clearly, such accuracy can only be achieved with explicit solvent and MD predictions of solvation energies (rather than implicit solvent methods), and it is our hope that the present study is a step in this direction.

## Supporting Information Available

Benchmarks of density and enthalpy of vaporization for ACN, DCE, water, and TCM with both OPLS-AA and SAPT-FF force fields; descriptions of OPLS-AA and SAPT-FF force fields for Fc/Fc^+^ and DMFc/DMFc^+^; implementation details for TI and linear response theory simulations; details on PBE0/COSMO calculations; and implementation details for computed interfacial potentials (PDF)
OpenMM simulation input files with force fields utilized in this work (ZIP)
