# Supplementary material for: Ion and Solvent Modulation of Ferrocene and Decamethylferrocene Oxidation Potentials in Organic Electrolytes as Predicted by Molecular Dynamics Simulations
Source: J Phys Chem B. 2025 Feb 11;129(7):2067–81. doi: 10.1021/acs.jpcb.4c08321 (PMC11848929; doi:10.1021/acs.jpcb.4c08321)
Supplement: Supplementary file 1 — jp4c08321_si_001.pdf [file jp4c08321_si_001.pdf]

Supporting Information for "Ion and solvent modulation of ferrocene and decamethylferrocene oxidation potentials in organic electrolytes as predicted by molecular dynamics simulations"

John H. Hymel, Suehyun Park, and Jesse G. McDaniel*

School of Chemistry and Biochemistry, Georgia Institute of Technology, Atlanta, Georgia 30332-0400, USA

E-mail: mcdaniel@gatech.edu

Contents

S1 Solvent Forcefield Benchmarks **S2**

S2 OPLS-AA Forcefield for Fc/Fc$^+$ **S3**

S3 SAPT-FF Forcefield for Fc/Fc$^+$ **S3**

S4 SAPT-FF Forcefield for DMFc/DMFc$^+$ **S6**

S5 Thermodynamic Integration **S7**

S5.1 Theory S7

S5.2 Implementation S8

S6 Linear Response Theory **S10**

S6.1 Theory S10

S6.2 Implementation S10

S7 Implicit Solvent Calculations **S11**

S8 Interfacial Potential Difference between Liquid and Vacuum for Bulk Solvents and Solvent/IL Mixtures **S12**

References **S13**

S1 Solvent Forcefield Benchmarks

To validate the force field parameters used in this study, the density and enthalpy of vaporization (ΔH_{vap}) of each solvent (acetonitrile (ACN), 1,2-dichloroethane (DCE), water (H_2O), chloroform (TCM)) was benchmarked against experimental data. This analysis was performed using both the OPLS-AA[1] and SAPT-FF[2–4] forcefields. OPLS-AA and SAPT-FF do not have their own explicit water models, and instead rely upon separately fit water models with which they are compatible. The TIP3P water model[5] is used with OPLS-AA and the polarizable SWM4-NDP water model[6] is used with SAPT-FF. Benchmarks for these various solvents are given in Table S1 and show good agreement with respect to experiment.

Table S1: Comparison of simulated and experimental densities and enthalpies of vaporization (ΔH_{vap}) for different solvents using OPLS-AA and SAPT-FF force fields.

**Solvent**	**Force Field**	**Density (g/cm^3)**			**ΔH_{vap} (kJ/mol)**		
		**sim.**	**exp.**	**% Error**	**sim.**	**exp.**	**% Error**
ACN	OPLS-AA	0.74	0.79	5.37	30.8	33.4	7.78
ACN	SAPT-FF	0.80	0.79	2.13	34.8	33.4	4.23
DCE	OPLS-AA	1.24	1.25	0.65	33.7	31.98	5.46
DCE	SAPT-FF	1.25	1.25	0.04	32.1	31.98	0.31
H_2O	TIP3P	1.00	1.00	1.43	42.5	43.9	3.22
H_2O	SWM4-NDP	1.00	1.00	0.16	43.7	43.9	0.48
TCM	OPLS-AA	1.50	1.49	0.69	30.2	31.4	3.98
TCM	SAPT-FF	1.49	1.49	0.21	31.4	31.4	0.13

S2 OPLS-AA Forcefield for Fc/Fc^+

The forcefield parameters for ferrocene (Fc) and ferrocenium (Fc^+) were refined from pre-existing models to accurately describe interactions within the various solvent environments. For OPLS-AA simulations, a modified form of the metallocene forcefield developed by Lopes et al.[7] was used. The original Lopes forcefield keeps the cyclopentadienyl rings internally rigid, this restriction was removed, and OPLS-AA aromatic carbon/hydrogen parameters were used to allow for flexibility of the ring. New charges were also parameterized for both Fc and Fc^+ using the approach of Ferenczy and co-workers.[8,9] This involved performing density functional theory calculations of Fc and Fc^+ at the PBE0/6-31G* level of theory, followed by electrostatic multipole fitting to a distributed multipole expansion analysis (DMA).[10,11] The forcefield parameters used for OPLS-AA simulations of Fc and Fc^+ are given in the tables of Figure S1.

S3 SAPT-FF Forcefield for Fc/Fc^+

To perform the simulations described in this paper, SAPT-FF forcefields were developed for both ferrocene (Fc) and ferrocenium (Fc^+). The OPLS-based forcefield outlined in section SS2 was used as a template for Fc and Fc^+, keeping the same bonded terms while fitting new SAPT-based nonbonded parameters. Reparameterization of the SAPT-FF forcefield was conducted using DFT(SAPT) calculations on approximately 2500 dimer configurations of Fc with acetonitrile (ACN) and 2500 dimer configurations of Fc with methane (CH4). The DFT(SAPT) interaction energies were computed using the Molpro software package.[12] Comparisons between the interaction energies calculated with the SAPT-FF for Fc and those from DFT(SAPT) showed good agreement, as illustrated in Figure S2.

During initial simulations with the Fc^+ forcefield, some configurations displayed solvent molecules positioned unrealistically close to the Fe center of Fc^+. This issue was traced to the absence of repulsive terms for the SAPT-FF Fe atom type. To address this,

Atom Type and Nonbonded Parameters						
Atom Type	mass (amu)	Fc charge (e)	Fc^+ charge (e)	σ (nm)	ϵ (kJ/mol)	Description
Fe	55.84	0.72	0.839	0.311	2.016	Iron center
Cr	12.0	-0.23225	-0.1766	0.355	0.293	Ring carbon
Hr	1.0	0.16025	0.1927	0.126	0.242	Ring hydrogen
Xcp	0.0	0.0	0.0	0.0	0.0	Ring center
Note: Xcp is a virtual site defined as the center of mass of the carbons within each cyclopentadienyl ring.						

Bond Parameters			
Type (i)	Type (j)	r (nm)	k (kJ/mol/nm^2)
Fe	Cr	0.2060	100000
Cr	Cr	0.1440	284500
Hr	Cr	0.1080	280000
Xcp	Xcp	0.3380	500000

Angle Parameters				
Type (i)	Type (j)	Type (k)	Angle (rad)	k (kJ/mol/radian^2)
Xcp	Fe	Xcp	3.14159	999.9
Hr	Cr	Cr	2.19911	292.9
Cr	Cr	Cr	1.88496	586.0

Improper Dihedral Parameters						
Type (i)	Type (j)	Type (k)	Type (l)	Periodicity	Phase (rad)	k (kJ/mol/radian^2)
Cr	Cr	Cr	Hr	2	3.14159	40.6

Figure S1: OPLS-AA Ferrocene Forcefield Parameters

an additional Born-Mayer-type repulsive potential was incorporated, based on the work of Ding et al.[13] Following this modification, further SAPT interaction energy benchmarks were conducted for a series of close-contact dimers, including Fc/ACN, Fc^+/ACN, Fc/H2O, Fc^+/H2O, Fc/trichloromethane (TCM), and Fc^+/TCM. These benchmarks were computed at the SAPT0/aug-cc-pVDZ level of theory using Psi4.[14] SAPT0 was chosen as it is the only SAPT code available that supports open-shell systems, such as Fc^+. The benchmarks comparing SAPT-FF and SAPT0 are presented in Figure S3, showing good agreement for

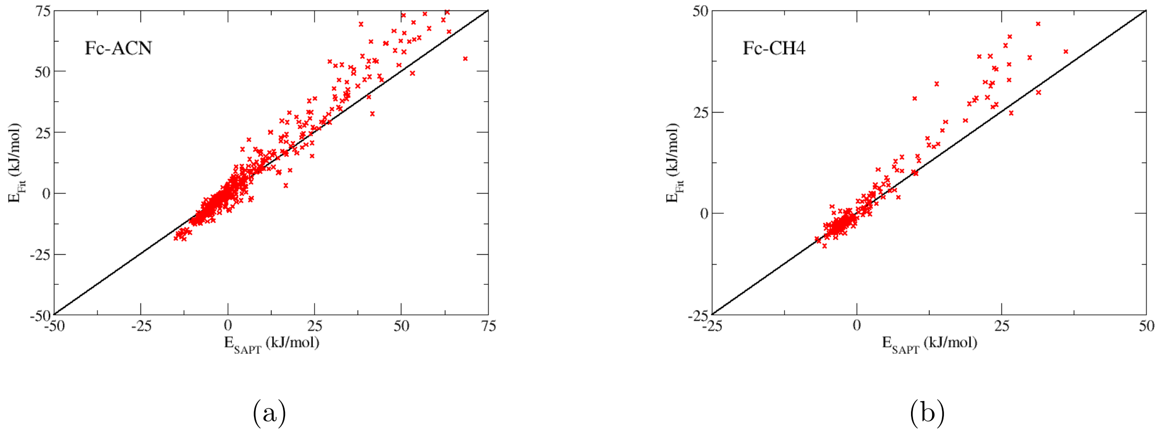

Figure S2: Comparison of SAPT-FF and DFT(SAPT) interaction energies computed for dimers of Fc/ACN and Fc/CH4.

Fc and reasonable agreement for Fc^+. The final nonbonded parameters fitted for SAPT-FF simulations of Fc and Fc^+ are provided in the tables in Figure S4.

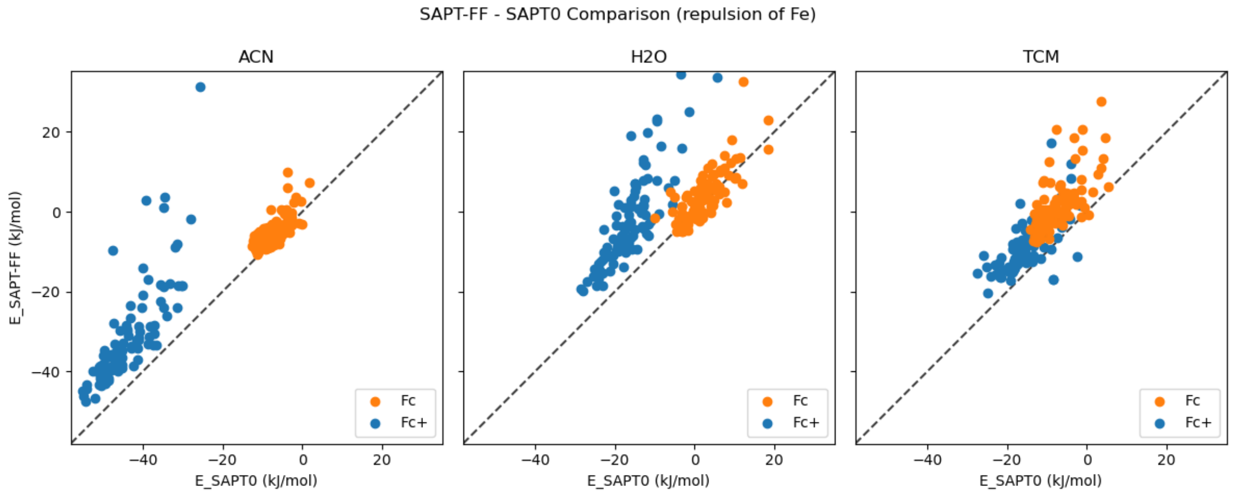

Figure S3: Comparison of SAPT0 and SAPT-FF interaction energies computed for close-contact dimers of Fc/Fc+ with ACN, H2O, and TCM.

Atom Type and Fit Charges					
Atom Type	mass (amu)	Fc charge (e)	Fc^+ charge (e)	Description	Polarizability
Fe	55.84	0.72	0.839	Iron center	N/A
Cr	12.0	0.91555	0.9712	Ring carbon	N/A
DCr	0.4	-1.1478	-1.1478	Ring carbon Drude	0.00195233
Hr	1.0	0.16025	0.1927	Ring hydrogen	N/A
Xcp	0.0	0.0	0.0	Dummy Ring center	N/A
Note: The charges listed here are identical to the static charges (q_s) used in the OPLS-AA simulations of Fc/Fc+. Charges listed here are q_{total}. For atoms with Drudes, i.e. Cr, $q_s = q_{total} + q_{Drude}$.					

SAPT-FF Nonbonded Parameters					
Atom Type	Aexch (kJ/mol)	Aelec (kJ/mol)	Aind (kJ/mol)	Adhf (kJ/mol)	Bexp (nm^{-1})
Fe	1.0e8	0.0	0.0	0.0	41.13
Cr	423935.1	204171.8	18740.5	44967.6	34.38
Hr	10600.4	2749.0	891.6	971.1	37.78
DCr	0.0	0.0	0.0	0.0	100.0
Xcp	0.0	0.0	0.0	0.0	100.0

SAPT-FF Additional Nonbonded Parameters				
Atom Type	C6 ($kJ/mol/nm^6$)	C8 ($kJ/mol/nm^8$)	C10 ($kJ/mol/nm^{10}$)	C12 ($kJ/mol/nm^{12}$)
Fe	0.0	0.0	0.0	0.0
Cr	1.68e-3	6.63e-05	6.42e-06	2.68e-07
Hr	1.34e-4	7.056e-06	5.186e-07	0.0
DCr	0.0	0.0	0.0	0.0
Xcp	0.0	0.0	0.0	0.0

Figure S4: SAPT-FF Ferrocene Forcefield Parameters.

S4 SAPT-FF Forcefield for DMFc/$DMFc^+$

To run simulations of decamethylferrocene (DMFc) and decamethylferrocenium ($DMFc^+$) using the SAPT-FF forcefield, additional parameters were needed beyond those developed for Fc and Fc^+. Specifically, bonded and nonbonded parameters were required for the added methyl groups. Standard bond, angle, and dihedral parameters for methyl groups were adopted from the OPLS-AA forcefield, while existing SAPT-FF parameters were used for the nonbonded interactions and Drude oscillator terms. Charges for DMFc and $DMFc^+$

were determined using the same procedure described in Section S2. For detailed forcefield parameters of DMFc and $DMFc^+$, refer to the OpenMM-compatible forcefield files included with this paper's supplemental information.

S5 Thermodynamic Integration

S5.1 Theory

Thermodynamic integration (TI) is used to compute the free energy difference between two different states, in this case, between two redox states. An alchemical coordinate, λ, is used to transition smoothly between initial and final states. Here, $\lambda = 0$ corresponds to the reduced state (ferrocene, Fc), and $\lambda = 1$ represents the oxidized state (ferrocenium, Fc^+). The difference in free energy between the two states is then defined via the following integral.

$$\Delta G^{TI} = \int_0^1 \left\langle \frac{\partial U(\vec{\Gamma}, \lambda)}{\partial \lambda} \right\rangle_\lambda d\lambda \tag{1}$$

In this equation, U is the potential energy of the system, $\vec{\Gamma}$ is a vector of all atomic coordinates, and $\langle ... \rangle_\lambda$ means that an ensemble average is taken over $\partial U / \partial \lambda$ at a fixed value of λ. The functional dependence of the potential energy on λ must be defined. For redox processes, if inner sphere reorganization is minimal, it can be assumed that the only difference between the reduced and oxidized states is in the fitted atomic charges, $\vec{q}$. In this case, varying λ involves linearly varying the charges of the redox molecules between those of the reduced and oxidized states.

$$\vec{q}(\lambda) = (1 - \lambda)\vec{q}_{red} - \lambda \vec{q}_{ox} \tag{2}$$

The derivatives described in equation S1 can then be computed numerically using a standard finite difference formula.

$$\frac{\partial U(\vec{\Gamma},\lambda)}{\partial \lambda} = \frac{U(\vec{\Gamma},\lambda+\Delta\lambda) - U(\vec{\Gamma},\lambda)}{\Delta\lambda} \tag{3}$$

While changing of charges of the redox molecule between reduced and oxidized states correctly samples the changing solvation environment, it also includes an artificial intramolecular component due to the change in intramolecular coulombic interactions. This effect is comparable to a forcefield-based ionization energy and is nonphysical. It can be computed as the difference in the average potential energy of the isolated redox molecule in the oxidized and reduced states.

$$\Delta E_{intra}^{redox} = \langle U_{ox} \rangle - \langle U_{red} \rangle \tag{4}$$

A choice needs to be made whether to treat electrostatics using a cutoff or PME in the short gas-phase simulations required to sample this potential energy difference. Using PME with the same unit cell size as used in the corresponding bulk liquid simulations would allow one to subtract off the interaction between the oxidized species and the background charge (due to the system being non-neutral). It has been shown by other researchers that leaving in interactions with the background charge corrects for finite size effects.[15] The solvation free energy difference between the oxidized and reduced states, computed using TI, is then defined by the following equation.

$$\Delta\Delta G_{solv}^{bulk,TI} = \Delta G^{TI} - \Delta E_{intra}^{redox} \tag{5}$$

S5.2 Implementation

In order to compute the $\Delta\Delta \mathrm{G}_{\mathrm{solv}}^{\mathrm{bulk,TI}}$ for Fc in bulk ACN, DCE, TCM, and H2O, as well as in mixtures of BMIM/BF4 with ACN and DCE, simulations were performed using the OpenMM software package. For each system, 6 simulations were run at $\lambda \in 0.0, 0.2, 0.4, 0.6, 0.8, 1.0$. Each simulation ran for a total of 100 ns per λ value with 1 Fc and 1000 solvent molecules.

$\partial U/\partial\lambda$ derivatives were computed numerically every 500 fs using a step size, $\Delta\lambda$, of 0.02. Integration with respect to λ was performed numerically using the trapezoid rule. When computing ΔE^{redox}_{intra}, cutoff-based electrostatics were used rather than PME in order to take advantage of finite-size corrections from the non-neutral simulation cell. For simulations utilizing the SAPT-FF forcefield, a dual-Langevin thermostat was used with the nuclei at 300K and Drude oscillators at 1K (as described in the methods section of the main text). When derivatives, $\partial U/\partial\lambda$, were computed, the positions of Drude particles were optimized using a Drude-SCF integrator, while the nuclei were kept fixed. This yielded derivatives absent of error associated with the thermal energy of the Drude particles. Benchmarks were performed to test how substantial the effect of thermalized Drudes was on $\partial U/\partial\lambda$ derivatives (Figure S5).

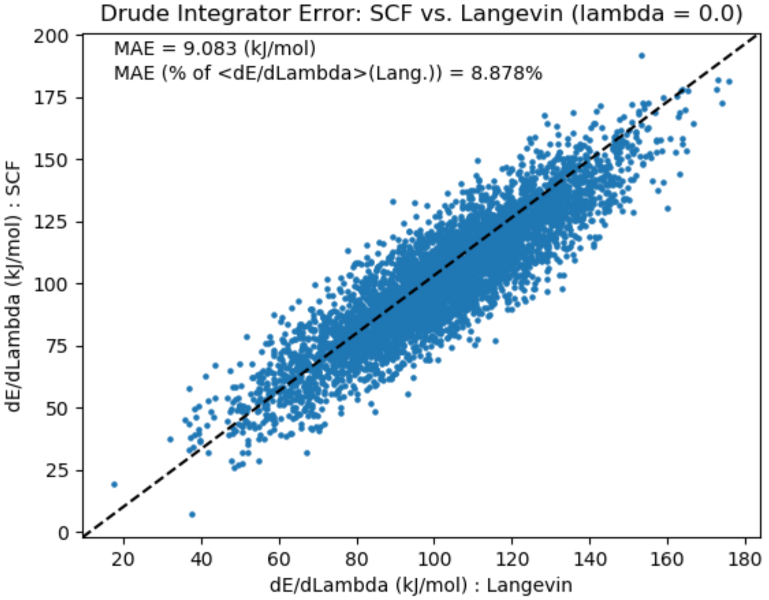

Figure S5: Benchmark comparing derivatives, $\partial U/\partial\lambda$, computed using Drude-Langevin and Drude-SCF at lambda = 0.0.

S6 Linear Response Theory

S6.1 Theory

Similar to thermodynamic integration (TI), linear response theory (LR) can be used to compute the free energy difference between two states, in this case, the reduced and oxidized states of a redox molecule. LR reduces the problem of computing the free energy difference to an average of the ensemble averages of the vertical energy gaps for each state.[16] The expression for the free energy difference computed using LR is given by equation S6:

$$\Delta G^{LR} = \frac{1}{2}\left[\langle \Delta V \rangle_{red} + \langle \Delta V \rangle_{ox}\right] \tag{6}$$

where $\Delta V = V_{ox} - V_{red}$, or more specifically, in the case of Fc/Fc$^+$, $\Delta V = V_{ion} - V_{neutral}$. LR offers computational benefits over TI as it requires simulations only at the endpoints, rather than for intermediate λ values between the physically meaningful states. Similar to TI, inner sphere reorganization is assumed to be small, and only the fitted atomic charges are varied between the oxidized and reduced states. The computed ΔG^{LR} also includes an artificial intramolecular contribution from the force field, ΔE^{redox}_{intra}, as discussed in the TI section of the Supporting Information. This contribution must also be accounted for in LR to compute the solvation free energy difference, $\Delta\Delta G^{bulk,LR}_{solv}$:

$$\Delta\Delta G^{bulk,LR}_{solv} = \Delta G^{LR} - \Delta E^{redox}_{intra} \tag{7}$$

S6.2 Implementation

To compute $\Delta\Delta G^{bulk,LR}_{solv}$ for Fc in mixtures of BMIM/BF4 with ACN and DCE, simulations were performed using the OpenMM software package. For each system, simulations were conducted at both the neutral and oxidized states. Each simulation ran for a total of 100 ns, with compositions corresponding to those given in Table 1 of the main text. Energy gaps,

ΔV, were computed every 10 ps. When computing ΔE_{intra}^{redox}, cutoff-based electrostatics were used instead of PME, to take advantage of finite-size corrections from the non-neutral simulation cell. These simulations utilized a dual-Langevin thermostat, maintaining the nuclei at 300 K and the Drude oscillators at 1 K (as described in the Methods section of the main text). During the computation of energy gaps, ΔV, the positions of Drude particles were optimized using a Drude-SCF integrator while keeping the nuclei fixed, yielding ΔV values without the thermal contribution from the Drude particles.

S7 Implicit Solvent Calculations

In the main text, estimations of the absolute oxidation potential, $\Delta G_{(sol)}^{Ox}$, are made using PBE0/lanl2dz and the "Conductor-like Screening Model" (COSMO) implicit solvation model.[17?] These calculations were performed using the NWChem quantum chemistry software package.[18] The absolute oxidation potential of ferrocene can be computed directly using DFT by taking the electronic energy difference between the neutral and oxidized ferrocene structures optimized in implicit solvent. This approach provides an oxidation potential with a DFT-level description of the ionization energy, which is highly dependent on the choice of density functional and challenging to compare to our TI results, which use an experimental ionization energy from NIST. To avoid these issues, we compute the differential solvation energy ($\Delta\Delta E_{solv}^{bulk}$), as shown in equation S8:

$$\Delta\Delta E_{solv}^{bulk} = (E_{COSMO}^{Fc^+} - E_{gas}^{Fc^+}) - (E_{COSMO}^{Fc} - E_{gas}^{Fc}) \tag{8}$$

were $E_{COSMO}^{Fc^+}$, $E_{gas}^{Fc^+}$, E_{COSMO}^{Fc}, and E_{gas}^{Fc} are the electronic energies of Fc or Fc^+, optimized in implicit solvent or the gas phase, respectively. This yields a DFT-level estimation of the differential solvation energy, which we then combine with the experimental ionization energy of Fc (from NIST) to compute the absolute oxidation potential ($\Delta G_{(sol)}^{Ox}$).

S8 Interfacial Potential Difference between Liquid and Vacuum for Bulk Solvents and Solvent/IL Mixtures

To extract the potential drop of the interface between the liquid and the vacuum from the solvation free energy, we compute the Poisson profile for the interfaces of the bulk solvent and the vacuum, and the solvent/[$BMIM^+$][BF_4^-] mixtures and the vacuum. MD simulations were employed for the bulk liquid periodic in two dimensions (x/y) with a larger than 10 nm vacuum gap to avoid the interaction with the periodic replicas in the z dimension. We use 1000 corresponding solvent molecules for bulk ACN, DCE, and TCM, and 2000 water molecules for bulk water. In the NVT ensemble, bulk solvents were initially equilibrated for 2 ns, followed by 18 ns of sampling. [$BMIM^+$][BF_4^-] were mixed with ACN and DCE and the exact system sizes are described in Table 1 of the main text. The solvent/IL mixtures were equilibrated for 10 ns and sampled for 90 ns due to the slow dynamics of ionic liquids. All liquid/vacuum interfacial potentials were calculated for nonpolarizable OPLS-AA and polarizable SAPT-FF force fields.

To determine the interfacial potential drop, it is necessary to compute the Poisson potential profile for the liquid/vacuum interface. Because of the symmetry in the xy plane, the Poisson profile is given by

$$\frac{d^2V(z)}{dz^2} = -4\pi\rho(z) \tag{9}$$

where $\rho(z)$ is the in-plane ensemble averaged charge density as a function of distance from the center of mass of the bulk liquid in the z dimension. Equation S9 is numerically integrated with a discretization of $\partial z = 0.05$ Å. The potential gap is determined by the difference in the potential values of the liquid and the vacuum region.

References

(1) Jorgensen, W. L.; Tirado-Rives, J. Potential energy functions for atomic-level simulations of water and organic and biomolecular systems. *Proceedings of the National Academy of Sciences of the United States of America* **2005**, *102*, 6665–6670.

(2) McDaniel, J. G.; Choi, E.; Son, C. Y.; Schmidt, J. R.; Yethiraj, A. Ab Initio Force Fields for Imidazolium-Based Ionic Liquids. *Journal of Physical Chemistry B* **2016**, *120*, 7024–7036.

(3) McDaniel, J. G.; Son, C. Y. Ion Correlation and Collective Dynamics in BMIM/BF4-Based Organic Electrolytes: From Dilute Solutions to the Ionic Liquid Limit. *The Journal of Physical Chemistry B* **2018**, *122*, 7154–7169.

(4) McDaniel, J. G.; Yethiraj, A. Influence of Electronic Polarization on the Structure of Ionic Liquids. *Journal of Physical Chemistry Letters* **2018**, *9*, 4765–4770.

(5) Price, D. J.; Brooks, I., Charles L. A modified TIP3P water potential for simulation with Ewald summation. *The Journal of Chemical Physics* **2004**, *121*, 10096–10103.

(6) Lamoureux, G.; Harder, E.; Vorobyov, I. V.; Roux, B.; MacKerell, A. D. A polarizable model of water for molecular dynamics simulations of biomolecules. *Chemical Physics Letters* **2006**, *418*, 245–249.

(7) Lopes, J. N.; Do Couto, P. C.; Da Piedade, M. E. An all-atom force field for metallocenes. *Journal of Physical Chemistry A* **2006**, *110*, 13850–13856.

(8) Chipot, C.; Angyan, J. G.; Ferenczy, G. G.; Scheraga, H. A. Transferable Net Atomic Charges from a Distributed Multipole Analysis for the Description of Electrostatic Properties - A Case-Study of Saturated-Hydrocarbons. *Journal of Physical Chemistry* **1993**, *97*, 6628–6636.

(9) Ferenczy, G. G. Charges Derived from Distributed Multipole Series. *Journal of Computational Chemistry* **1991**, *12*, 913–917.

(10) Stone, A. J.; Alderton, M. Distributed Multipole Analysis - Methods and Applications. *Molecular Physics* **1985**, *56*, 1047–1064.

(11) Stone, A. J. Distributed Multipole Analysis: Stability for Large Basis Sets. *Journal of Chemical Theory and Computation* **2005**, *1*, 1128–1132.

(12) Werner, H.-J. et al. The Molpro quantum chemistry package. *The Journal of Chemical Physics* **2020**, *152*, 144107.

(13) Ding, F.; Bolton, K.; Rosén, A. Iron-carbide cluster thermal dynamics for catalyzed carbon nanotube growth. *Journal of Vacuum Science Technology A* **2004**, *22*, 1471–1476.

(14) Smith, D. G. A. et al. PSI4 1.4: Open-source software for high-throughput quantum chemistry. *J. Chem. Phys.* **2020**, *152*, 184108.

(15) Hummer, G.; Pratt, L. R.; García, A. E. Free Energy of Ionic Hydration. *The Journal of Physical Chemistry* **1996**, *100*, 1206–1215.

(16) Aqvist, J.; Hansson, T. On the validity of electrostatic linear response in polar solvents. *Journal of Physical Chemistry* **1996**, *100*, 9512–9521.

(17) Klamt, A.; Schüürmann, G. COSMO: a new approach to dielectric screening in solvents with explicit expressions for the screening energy and its gradient. *Journal of the Chemical Society, Perkin Transactions 2* **1993**, 799–805.

(18) Aprà, E. et al. NWChem: Past, present, and future. *The Journal of Chemical Physics* **2020**, *152*, 184102.
